# Selection Transforms the Landscape of Genetic Variation Interacting with Hsp90

**DOI:** 10.1371/journal.pbio.2000465

**Published:** 2016-10-21

**Authors:** Kerry A. Geiler-Samerotte, Yuan O. Zhu, Benjamin E. Goulet, David W. Hall, Mark L. Siegal

**Affiliations:** 1 Center for Genomics and Systems Biology, Department of Biology, New York University, New York, New York, United States of America; 2 Department of Biology, Stanford University, Stanford, California, United States of America; 3 Department of Genetics, Stanford University, Stanford, California, United States of America; 4 Department of Genetics, University of Georgia, Athens, Georgia, United States of America; Fred Hutchinson Cancer Research Center, United States of America

## Abstract

The protein-folding chaperone Hsp90 has been proposed to buffer the phenotypic effects of mutations. The potential for Hsp90 and other putative buffers to increase robustness to mutation has had major impact on disease models, quantitative genetics, and evolutionary theory. But Hsp90 sometimes contradicts expectations for a buffer by potentiating rapid phenotypic changes that would otherwise not occur. Here, we quantify Hsp90’s ability to buffer or potentiate (i.e., diminish or enhance) the effects of genetic variation on single-cell morphological features in budding yeast. We corroborate reports that Hsp90 tends to buffer the effects of standing genetic variation in natural populations. However, we demonstrate that Hsp90 tends to have the opposite effect on genetic variation that has experienced reduced selection pressure. Specifically, Hsp90 tends to enhance, rather than diminish, the effects of spontaneous mutations and recombinations. This result implies that Hsp90 does not make phenotypes more robust to the effects of genetic perturbation. Instead, natural selection preferentially allows buffered alleles to persist and thereby creates the false impression that Hsp90 confers greater robustness.

## Author Summary

Most biologists appreciate that natural selection filters new mutations (e.g., by eliminating deleterious ones), such that genetic variation in nature is biased. The idea that selection also skews the types of genetic interactions that exist in nature is less appreciated. For example, studies spanning diverse species have shown that the protein Hsp90, which helps other proteins to fold properly, tends to diminish the observable effects of genetic variation. This observation has led to the assumption that Hsp90 also buffers the effects of new mutations. This untested assumption has served as a rationale for cancer-treatment strategies and shaped our understanding of variation in complex traits. We measured the effects of new mutations on the shapes and sizes of individual yeast cells and found that Hsp90 does not tend to buffer these effects. Instead, Hsp90 interacts with new mutations in diverse ways, sometimes buffering, but more often enhancing mutational effects on cell shape and size. We conclude that selection preferentially allows buffered mutations to persist in natural populations. This result alters common perceptions about why cryptic (i.e., buffered) genetic variation exists and casts doubt on cancer-treatment strategies aiming to target presumed buffers of mutational effects.

## Introduction

**Fig 1:**
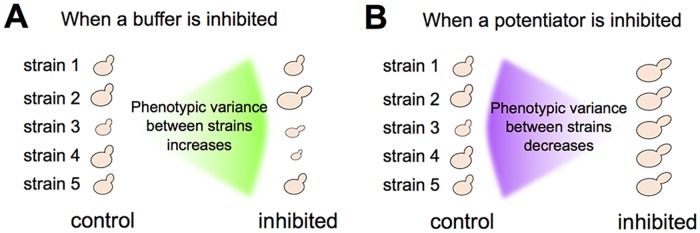
Responses of genetically diverse populations to inhibition of a buffer or potentiator. (A) Inhibiting a protein that buffers the phenotypic effects of genetic variation will reveal those effects, which increases the phenotypic diversity between genetically distinct individuals. (B) Inhibiting a protein that potentiates the phenotypic effects of genetic variation will diminish those effects, which decreases the phenotypic diversity between genetically distinct individuals.

Previous work in diverse eukaryotes has demonstrated that inhibition of the protein-folding chaperone Hsp90 reveals previously hidden phenotypic effects of standing genetic variation (see “buffering” in Fig 1A) [1–6]. This result prompts an important question [7–10]: does Hsp90 increase an organism’s robustness to genetic perturbation? That is, by helping mutant proteins to fold, does Hsp90 buffer the phenotypic consequences of many new mutations that would otherwise have effects? An alternative possibility is that Hsp90-buffered mutations are rare, yet appear more prevalent in nature because stabilizing selection does not purge such mutations, whereas it efficiently purges other mutations that have immediate (non-buffered) effects on phenotype [7].

Understanding robustness is crucial for understanding evolutionary processes [10–12] as well as complex disease (i.e., why certain mutations are associated with disease states in some genetic backgrounds but not others) [13]. Furthermore, the potential to modulate robustness by inhibiting Hsp90 presents an intriguing strategy to treat cancer [14–16]; because tumors have increased mutation rates, they might be particularly reliant on proteins that buffer the effects of mutation [17,18]. Although discussions of mutational robustness (sometimes termed genetic canalization) often feature Hsp90 [5,15,19–24], the aforementioned hypothesis that Hsp90-buffered variation is rare but accumulates in natural populations due to selection [7–10] has not been excluded.

There is evidence that Hsp90 sensitizes cells to, rather than buffers, the effects of some genetic perturbations (see “potentiation” in Fig 1B) [6,25,26]. Genetic variants with effects that are more pronounced when Hsp90 is present are particularly common in studies of populations that recently underwent adaptive evolution [25,26], reinforcing the hypothesis that natural selection filters which types of Hsp90-by-genotype interactions exist in nature. To distinguish the “robustness” hypothesis from the “selection” hypothesis, we study how Hsp90 modifies the phenotypic effects not only of standing genetic variation but also of new mutations that have experienced highly reduced selection pressure.

## Results

### A Collection of Mutations That Experienced Reduced Selection Pressure

To study new mutations, we utilized 94 *Saccharomyces cerevisiae* mutation accumulation (MA) lines created previously [27]. These yeast lines were all founded by the same ancestral diploid strain. To produce each MA line, independent derivatives of this ancestral strain were bottlenecked 100 times each through single colonies that were randomly chosen—irrespective of colony size—in order to relax selection on growth rate and other fitness-related traits [27]. Because the lines were propagated asexually, recessive mutations were shielded as heterozygotes, further relaxing selection. During the repeated bottlenecking, which was carried out for an estimated 2,062 generations [27], each line accumulated on average ~4 single-nucleotide mutations per haploid genome as well as a greater number of mutations in simple-sequence repeats [28,29]. The single-nucleotide mutations do not show a signature of selection; for example, the ratio of synonymous to non-synonymous substitutions does not deviate from random expectation [28].

A previous study demonstrated that the MA lines contain mutations that affect the morphologies of individual cells [9]. As in the previous study, we used haploid derivatives of the MA lines to assay the effects of mutations absent any influence of dominance [9]. Our expectation was that many of these lines would contain mutations with Hsp90-dependent effects on single-cell morphology. Although Hsp90 mediates the folding of only a subset of “client” proteins [30], Hsp90 interactions may be indirect, mediated through other proteins connected to Hsp90 via a protein-protein network.

### A Quantitative Technique for Detecting Buffering and Potentiation

**Fig 2:**
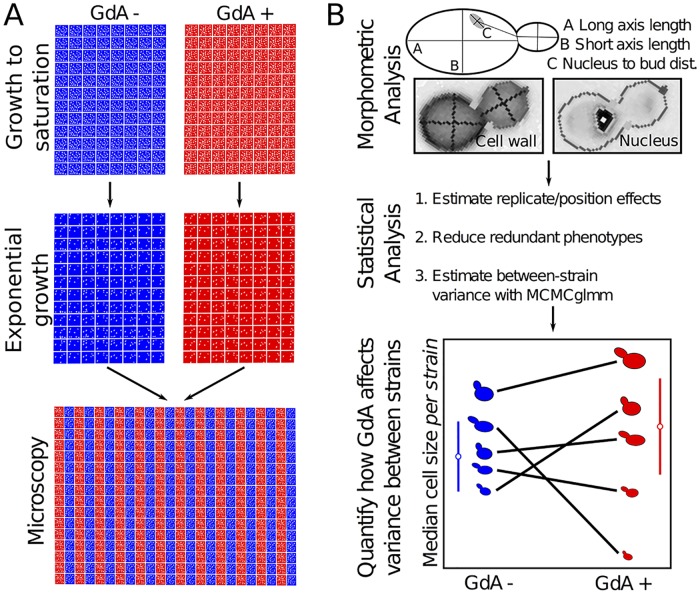
Experimental design. This figure outlines the experimental (A) and analytical (B) procedures used to quantify variation in morphological traits with and without Hsp90 inhibition (see S1 Text and S1 Fig).

We used high-throughput microscopy and automated image analysis [31] to quantify how Hsp90 inhibition changes variation in single-cell morphology between MA lines. We quantified differences in morphological variation using a statistical approach [9] that detects significant increases or decreases in phenotypic variance upon Hsp90 inhibition (corresponding, respectively, to buffering or potentiating roles of Hsp90). Detecting changes in phenotypic variance that are specifically due to Hsp90 inhibition requires an experimental design (Fig 2) that accounts for the other factors that might affect phenotypic variation. Measuring phenotypic variance is far less common than studying phenotypic averages and presents a unique set of challenges [32]. Therefore, we describe our approach in some detail.

We inhibited Hsp90 using geldanamycin (GdA), a small-molecule inhibitor that binds the ATP-binding site of Hsp90, rendering it unable to perform its cellular function [33]. This drug is commonly used to inhibit Hsp90 in diverse species [1–4], including yeast [6,34]; GdA and its analogs have also been administered in human clinical trials to treat cancers [14,35,36]. Previous studies of Hsp90-mediated buffering in yeast used GdA concentrations ranging from 5 μM [6] to 200 μM [34]. We performed the majority of our experiments at 8.5 μM GdA because this concentration has minimal effects on the growth rate and lag duration of the yeast strains we study (S1 Fig).

In each step (cell growth, cell staining, microscopy) of each replicate experiment in this study, cells grown in Hsp90-inhibited (8.5 μM GdA) and control conditions were studied side by side (Fig 2A). The effects of GdA are therefore not confounded with replicate effects, which we estimated and removed using linear modeling. In each replicate, cells were grown to saturation followed by exponential growth in 96-well plates for 6 h to a density of approximately 1 x 10^6^ cells/mL. Then cells were fixed in 4% paraformaldehyde and stained with dyes that label cell-wall components and nuclear DNA. Stained cells grown in control or inhibited conditions were mounted side by side, in duplicate, on 384-well microscopy plates. High-throughput microscopy and morphometric analysis [31] typically yielded between 100 and 500 quantified cell images from each cell-cycle stage in each strain in each replicate experiment (S1 Fig).

In addition to variation between replicate experiments, there is another source of variation that must be considered when estimating phenotypic variance between yeast strains: cell-to-cell variation within each yeast strain. Hsp90 inhibition has been shown to increase nongenetic heterogeneity for some single-cell morphological features [34], so the common statistical assumption of homogeneous within-group variances is likely to be violated. Therefore, for each phenotype, we partitioned the within-strain variation and between-strain variation, as in previous work [9], with separate within-strain variance terms for Hsp90-inhibited and control conditions by using a Bayesian mixed-model approach based on Markov chain Monte Carlo (MCMC) sampling, implemented using the R package MCMCglmm [37].

Using the approach outlined above (and in Fig 2), we quantified between-strain variation in 29 principal components (PCs) of MA-line morphology that capture most of the variance of 132 single-cell traits. These 132 traits represent a high-quality group of morphometric features identified in a previous study as having lower variation across replicate experiments [38]. Each trait is specific to cells from a particular phase of cell growth, such that 6 PCs represent 83% of the variance in morphology among yeast cells without a bud, 9 PCs represent 85% of the variance among cells with a small bud, and 14 PCs represent 86% of the variance among cells with a large bud (S1 Table). Our ultimate goal was, for each of these PCs (hereafter “phenotypes”), to assess whether Hsp90 inhibition significantly increases or decreases the amount of phenotypic variation between MA lines (as schematized in Fig 1). But first, we asked more generally whether the effects of the mutations in the MA lines are influenced by Hsp90 (without asking whether such mutations are buffered or potentiated).

### MA Lines Possess Mutations with Hsp90-Dependent Effects on Single-Cell Morphology

**Fig 3:**
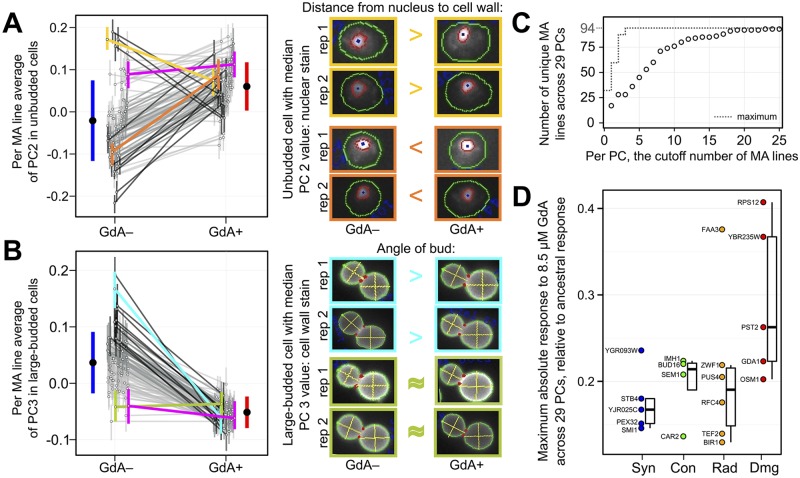
Many MA lines possess spontaneous mutations with GdA-dependent effects on morphology. (A–B) Two example phenotypes for which some MA lines have unique responses to GdA (for other phenotypes, see S2 Fig). Each open circle and its associated vertical bar represents the average morphology of an individual MA line in either the GdA− or GdA+ condition, +/– 1 standard deviation. Lines connect open circles representing the same MA line; the darkness of each black line is proportional to how much a given MA line’s response to GdA differs from the ancestor’s. Colored lines: magenta = MA ancestor; yellow = MA line #2; orange = MA line #30; cyan = MA line #42; olive = MA line #137. Micrographs (with cell images adjusted for uniform size and orientation) reflect how morphological traits that contribute strongly to each PC respond to GdA in selected strains. The cells shown from each replicate (“rep 1” or “rep 2”) possess the value closest to the median for a given PC in the given strain and treatment. The trait that contributes most strongly to each PC is listed above the cell images (and in S1 Table); the >, <, or ≈ symbols indicate how this trait differs in GdA− versus GdA+ cells. Each solid black circle and its associated blue or red vertical bar reports the average phenotype +/– 1 standard deviation across all MA lines in GdA− or GdA+. (C) The MA lines with the most divergent responses to GdA, relative to the ancestral response, differ for different PCs (see also S3 Fig and S2 Table). This cumulative distribution describes the number of unique MA lines represented among the top 1 through 25 most-divergently responding MA lines for each PC (open circles). The dotted line shows the maximum possible value. (D) MA lines possessing coding mutations predicted to have severe effects on protein function tend to have greater responses to GdA relative to the ancestral response (see also S2 Table). Open circles represent 20 of the 94 MA lines that each possess only a single coding mutation. The mutated gene is listed next to the circle. Vertical axis represents the maximum absolute value of the difference in MA line versus ancestral response to GdA across all 29 PCs. Boxplots represent the distribution of these maximum absolute differences for MA lines with either a single synonymous (“Syn”), conservative (“Con”), radical (“Rad”), or damaging (“Dmg”) mutation, displaying the median (center line), interquartile range (IQR) (upper and lower hinges), and highest value within 1.5 × IQR (whiskers).

Several observations suggest that the MA lines possess mutations with effects on morphology that differ when cells are grown in the control versus the 8.5 μM GdA condition (Fig 3). Linear modeling detects significant condition-by-line interactions contributing to variation in 27/29 phenotypes (and 108/132 of the original traits) at *p* < 0.001 (S1 Table).

These statistically detected interactions are borne out by inspection of cell images (Fig 3A and 3B). For example, the second PC in unbudded cells is strongly influenced by traits that concern the position of the nucleus (see loadings in S1 Table). The average distance from the nucleus to the cell wall in the MA line ancestor does not significantly change upon GdA treatment (magenta line in Fig 3A). However, in some MA lines, the average distance from the nucleus to the cell wall decreases upon GdA treatment (e.g., yellow line and cell images in Fig 3A); in other MA lines, the average distance from the nucleus to the cell wall increases upon GdA treatment (e.g., orange line and cell images in Fig 3A). A similar example is presented by the third PC in large-budded cells, which is strongly influenced by the bud angle. Whereas the average bud angle in the MA line ancestor (magenta line in Fig 3B) and in several MA lines (e.g., olive line in Fig 3B) does not change significantly upon GdA treatment, the average bud angle in other MA lines does (e.g., cyan line in Fig 3B). Both examples make clear that mutations in the MA lines have effects that differ between the control and GdA treatments; results from other phenotypes are consistent with this conclusion as well (S2 Fig).

The significant interactions are not all driven by a handful of MA lines possessing mutations with particularly strong GdA-dependent effects. Rather, multiple MA lines demonstrate GdA-induced phenotypic changes that diverge from those observed in the MA ancestor (Fig 3A and 3B; S3 Fig), so much so that there are 17 distinct most-divergent MA lines across 29 PCs (Fig 3C).

Our finding that many MA lines undergo phenotypic changes upon GdA treatment that diverge from those of the MA ancestor suggests that the phenotypic effects of many mutations are influenced by Hsp90. It is unlikely that the few mutations per each MA line mostly fall in the coding sequences of Hsp90 clients. Further study of the mutations in MA lines with GdA responses that diverge from those of the ancestor could reveal whether Hsp90 often interacts directly with mutant versions of non-client proteins, or whether Hsp90’s influence on mutant phenotypes often manifests through indirect interactions percolating through protein networks [39,40]. Because most MA lines possess multiple mutations, we do not know which ones are responsible for the divergent GdA responses. Therefore, to gain insights about what types of mutations have GdA-dependent phenotypic effects, we focused on a subset of 20 MA lines for which whole genome sequencing identified only one single-nucleotide mutation each (S2 Table). Of these MA lines, those that possess mutations predicted to have severe effects on protein function (S2 Table) [28,41] tend to have responses to GdA treatment that diverge more from those of the MA ancestor (Fig 3D).

### GdA-By-Genotype Interactions Are Dominated by Line-Crossing Epistasis

**Fig 4:**
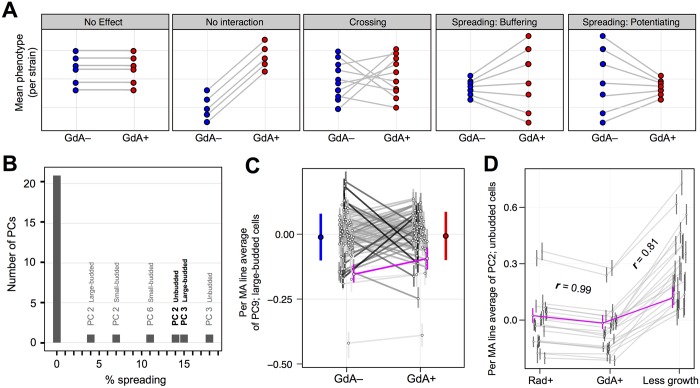
Epistatic interactions between Hsp90 and spontaneous mutations do not often involve buffering or potentiation. (A) Models of the different effects that Hsp90 might have on phenotypic variation between strains are shown. Each plot displays median phenotype per strain in GdA− (blue circles) and GdA+ (red circles); each line connecting two circles follows the change that Hsp90 inhibition has on a given strain. When lines have different slopes, GdA has a genotype-specific effect (rightmost 3 models). Line-crossing epistasis can be distinguished from buffering or potentiating, which are line-spreading subtypes of epistasis. (B) Line-crossing epistasis is far more prevalent than buffering or potentiation between Hsp90 and the spontaneous mutations present in the MA lines. The horizontal axis represents the fraction of the interaction between GdA and genotype that can be explained by line spreading (as opposed to line crossing); the vertical axis represents the number of PCs that fall into each bin, where bin width is 1%. PCs for which line spreading contributes >1% of this interaction are labeled; bold-labeled PCs are those plotted in Fig 3A and 3B. (C) An example phenotype for which line crossing contributes >99% (spreading contributes <1%) of the interaction between GdA and MA lines. These data are plotted as in Fig 3A. (D) The effects of GdA on MA line morphology are more similar to the effects of another Hsp90 inhibitor radicicol (Rad) than they are to the effects of abbreviated growth. Displayed plots are similar to those in Fig 3A, except here they show a random subset of 22 MA lines that were imaged after growth in GdA, Rad, and in a “less growth” condition in which we reduced the duration of exponential phase from 6 to 4 h (see *Experimental Procedures*). This PC was chosen because both correlation coefficients (***r***) are close to their median values across all 29 PCs (for all PCs, see S4 Fig).

GdA-by-genotype interactions can be partitioned [9] into a component that captures changes in the amount of phenotypic variance between MA lines (Fig 4A; line spreading) and a component that captures changes in rank order of MA lines (Fig 4A; line crossing). Such partitioning quantifies the importance of buffering or potentiation (line spreading) relative to genetic interactions that do not alter the phenotypic variance among lines (line crossing). For all 27 PCs with a significant condition-by-line interaction term in linear models, line spreading comprises only a relatively small percentage of Hsp90’s effect on phenotypic variation between strains; instead, line crossing dominates (Fig 4B; for a PC demonstrating the typical ratio of line crossing to line spreading, see Fig 4C). The strong prevalence of line crossing across all PCs suggests that Hsp90 rarely acts specifically as a buffer or a potentiator, but often its relationship with spontaneous mutations can be described more accurately (and simply) as epistasis [10]. These observations, by suggesting that Hsp90 does not act exclusively—or even usually—as a buffer of spontaneous mutations, do not support the hypothesis that Hsp90 increases an organism’s robustness to genetic perturbation.

Assuming that GdA treatment is equivalent to inhibition of Hsp90, the above observations suggest there is epistasis between Hsp90 and many mutations that occur spontaneously in nature. This conclusion is consistent with Hsp90’s position as a hub of protein-protein interaction (because hub proteins, either directly or indirectly, likely influence the phenotypic effects of mutations in many other proteins). However, there might be other reasons that GdA treatment affects phenotypic variation, including reduction of growth. Although previous studies [1,2,6] concluded that the effects of low-level GdA on phenotypic variation result from Hsp90 inhibition and not a nonspecific effect of GdA on growth, and although 8.5 μM GdA has minimal effects on growth of the strains we study (S1 Fig), we sought to test this assumption as well.

We performed additional experiments that indeed support the conclusion that GdA treatment affects phenotypic variation through its inhibition of Hsp90. We find that the MA line responses to GdA and to 5.0 μM radicicol (another Hsp90 inhibitor that is structurally unrelated to GdA [42]) are highly correlated; the median Pearson correlation across 29 PCs (***r***) is 0.95 (Fig 4D; for all PCs, see S4 Fig). MA line morphological responses to altering the duration of exponential growth are not as correlated (median ***r*** = 0.76) (Fig 4D; S4 Fig). Furthermore, although altering exponential growth duration often has a significant effect on the parameter we are most interested in—the amount of between-strain variation (S5A Fig)—neither the magnitude nor the direction of this effect predicts which phenotypes have between-strain variances that are significantly influenced by GdA (S5B Fig). Finally, as noted below, restricting our dataset to traits for which variation is not significantly affected by growth duration does not alter the conclusions reported for the analyses of between-strain variation in the MA lines and other strain collections. These results suggest that GdA’s influence on between-strain phenotypic variation does not result generally from growth perturbation but rather is a specific consequence of Hsp90 inhibition.

### Hsp90 Does Not Increase Mutational Robustness

**Fig 5:**
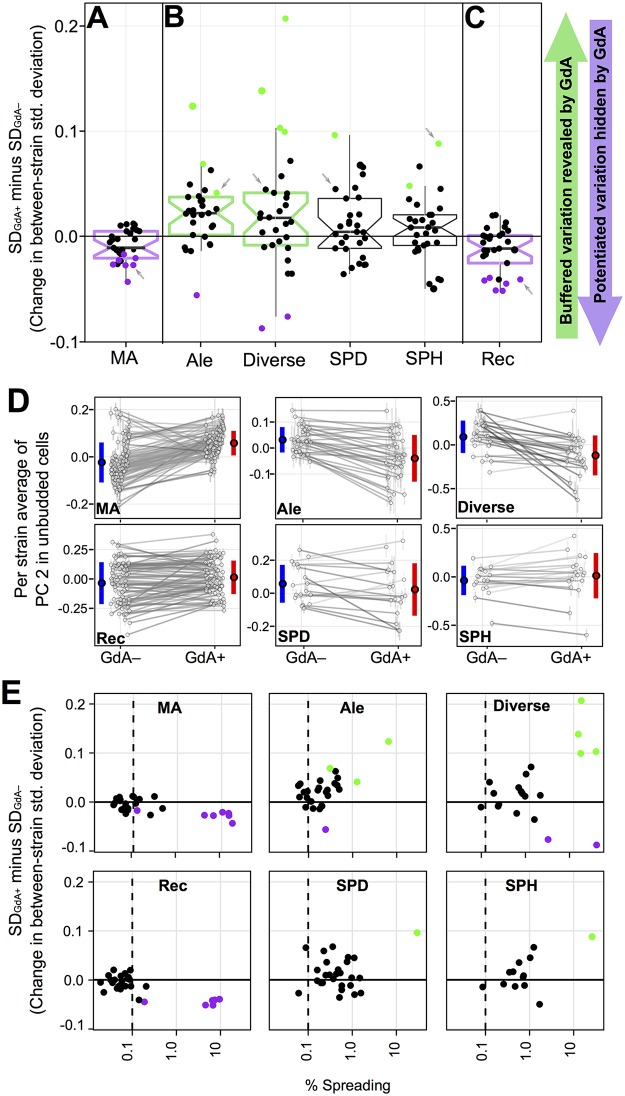
GdA’s effect on morphological variation differs in yeast strains possessing spontaneous mutations or recombinations, as compared to natural yeast isolates. In (A–C), each point is plotted to represent between-strain morphological variation (standard deviation) in the GdA+ condition minus that in the GdA− condition; colored dots represent significant variance increases (green) or decreases (purple) in GdA+ versus GdA− (significance defined as when the 95% credible interval surrounding the difference does not overlap zero). Boxplots summarize the distribution across all 29 PCs for each strain collection, displaying the median (center line), interquartile range (IQR) (upper and lower hinges), highest value within 1.5 × IQR (whiskers), and roughly a 95% confidence interval around the median calculated as 1.58 × IQR / √n (notches). If this confidence interval does not overlap zero, boxplots are colored green when Hsp90-inhibition tends to reveal variation and purple when inhibition tends to hide variation. Although PCs differ in the amount of variance explained, each is scaled to have an overall variance of 1. Panels represent GdA’s effect on (A) variation between MA lines, (B) variation between strains in four collections of yeast isolated from natural environments, and (C) variation between recombinant progeny of a mating between two divergent yeast strains (see S5 Fig for similar plots depicting only those PCs for which variance is not affected by growth perturbations). (D) For the PC indicated by grey arrows in panels A–C, these plots display the average morphologies for each strain in GdA+ and GdA− conditions as well as the between-strain variation (blue and red bars), which decreases in GdA+ for MA and Rec lines but increases in other strain collections. Plots are drawn as in Fig 3A (for all PCs, see S2 Fig). (E) Points represent only those PCs that have a significant GdA-by-genotype interaction in linear models for each strain collection (see S1 Table). The horizontal axis represents the fraction of this interaction that can be explained by line spreading (as opposed to line crossing). The dashed line helps guide the eye to see that this fraction, although low across all strain collections, is lowest for those that experienced reduced selection pressure (MA and Rec) relative to collections of natural yeast isolates (Ale, Div, SPD, and SPH; for additional evidence that natural isolates experienced stabilizing selection on morphological traits, see S6 Fig). The vertical axis represents the same as in panels A–C: the between-strain morphological variation (standard deviation) in the GdA+ condition minus that in the GdA− condition. Points are colored as in panels A–C.

Although GdA treatment impacts the phenotypes of individual MA lines more than it changes the overall amount of variance between lines (Fig 4B), we can still quantify whether such variance changes tend more toward buffering or potentiation. If yeast cells are robust to the effects of spontaneous mutations in a way that is compromised by Hsp90 inhibition, we would expect GdA treatment to increase phenotypic diversity between MA lines by revealing the previously buffered effects of mutation (as schematized in Fig 1A). Instead, for many phenotypes, GdA treatment decreases morphological variation between MA lines (compare ranges of blue and red lines in Fig 3A and 3B). To determine if these differences in variance are significant, we asked whether a 95% credible interval around each difference overlaps zero. Using this cutoff, several phenotypes (7 of 29 PCs; Fig 5A; S1 Table) demonstrate significant decreases in variance upon GdA treatment. Using the same cutoff, no phenotype displays a significant increase in between-strain variance. A 95% confidence interval surrounding the median of the variance differences across all 29 PCs (Fig 5A) falls entirely below zero, suggesting that inhibiting Hsp90 with GdA generally tends to decrease the phenotypic effects of mutations on MA-line morphologies. These observations imply that Hsp90, when not inhibited, tends to potentiate rather than buffer the phenotypic effects of new mutations. This conclusion is inconsistent with the “robustness” hypothesis described above.

The idea that Hsp90 increases robustness against the effects of new mutations has persisted [5,14,15,19–24] despite alternate models for the prevalence of buffered variation in natural populations [7,8], despite an absence of studies focusing on new mutations rather than standing genetic variation [7,43], and despite the previous observations that in some cases Hsp90 potentiates, rather than buffers, the effects of genetic perturbation [6,25,26]. Our finding that Hsp90 tends to potentiate the effects of new mutations more than to buffer them (and that neither potentiation nor buffering is nearly as salient as line-crossing epistasis) must be reconciled with the observation, made in species spanning eukaryotic diversity, that impairing Hsp90 reveals cryptic genetic variation [1–6]. We investigated the hypothesis that, although buffered mutations are less likely to arise than potentiated mutations, buffered mutations preferentially accumulate in populations over evolutionary time [7].

### Hsp90 Inhibition Tends to Increase Variation between Natural Yeast Isolates

To test this selection hypothesis, we repeated all experiments on several additional collections of yeast strains isolated from natural environments (S3 Table). These four yeast collections, hereafter called the “Ale,” “Diverse,” “SPD,” and “SPH” strains, consist respectively of (1) 36 strains used in ale production; (2) 24 strains from a worldwide collection from diverse habitats [44,45]; (3) 18 strains of the closely related, nondomesticated species *Saccharomyces paradoxus*, isolated from one soil environment in England [44,45]; and (4) 18 haploid derivatives of the SPD strains [45].

Unlike the mutations in the MA lines, which accumulated under artificially reduced selection pressure, the standing genetic variation among natural yeast isolates has survived natural selection. Patterns of genetic variation in the collections of natural isolates that have been sequenced (Diverse, SPH, and SPD) reveal evidence of selection [44,46]. These studies also suggest that fermentation processes, like those used to cultivate strains in the Ale collection, impose selective pressures [44,46]. Further studies demonstrate that stabilizing selection has acted specifically on the morphological traits that we study, constraining morphological variation between strains in the Diverse collection [47]. Consistent with strong stabilizing selection, we find that levels of morphological variation in each of the four collections of natural isolates do not far exceed those observed in the MA lines (S6 Fig), even though their levels of genetic diversity are vastly greater [44]. These observations, plus the strong relationship between yeast morphology and critical processes such as growth and reproduction [47], provide compelling evidence that genetic variation contributing to single-cell morphology in the Ale, Diverse, SPD, and SPH strain collections has been constrained by natural selection.

The selection hypothesis predicts that the single-cell morphologies in yeast collections representing natural isolates will tend to become more divergent upon Hsp90 inhibition. Consistent with the selection hypothesis, Hsp90 inhibition tends to increase morphological diversity in all four such yeast collections (Fig 5B). The qualitatively different responses to GdA observed in the natural isolates versus the MA lines persist when the MA lines are subsampled (S5A Fig), when we eliminate phenotypes for which between-strain variation is influenced by growth (S5C Fig), and when we study separately those phenotypes specific to cells in different phases of the cell cycle (S5D Fig). The tendency toward potentiation in the MA lines also persists when we restrict our analysis to the 20 MA lines that each possess a single coding mutation (S5E Fig), arguing against the possibility that this tendency is somehow caused by the combined effects of multiple spontaneous mutations per line.

### Hsp90 Inhibition Tends to Decrease Variation between Lines with Novel Genetic Recombinations

The distinction between the MA lines and natural isolates might support the hypothesis that natural selection acts as a filter of new mutations with epistatic interactions [7,8], skewing the relationship between Hsp90 and the rest of the genotype from one that tends toward potentiation to one that tends toward buffering. However, another possibility is that the ancestor of the MA lines possesses a specific genetic make-up that causes it to interact differently with GdA. Perhaps MA-line growth is uniquely responsive to GdA in a way we did not control for. Or perhaps the MA line ancestor possessed a faulty copy of a gene involved in protein homeostasis that causes Hsp90 inhibition to have unexpected effects. To rule out such possibilities, we repeated our experiments on an additional yeast strain collection that consists of 78 recombinant (Rec) progeny from a cross between two divergent yeast strains from the “Diverse” collection [48]. Although each individual mutation in these Rec lines has been exposed to selection, the unique combination of mutations in each line has not, because the mutations are largely private to one or the other lineage [44]. Therefore, studying these lines served two purposes: (1) testing an additional collection of lines each representing a unique genotype that has not experienced selection and (2) testing if Hsp90 buffers the effects of recombination, as it has been suggested that recombination exerts a selective force favoring genetic buffering [49,50].

Previous studies of Rec lines have produced mixed results. A study in yeast found roughly equal frequencies of buffering and potentiation [6]. In contrast, studies in plants concluded that genetic buffering by Hsp90 is common [2,4,20]. However, in the plant Rec inbred lines, line-specific phenotypes revealed by Hsp90 inhibition were also seen in those lines without Hsp90 inhibition, albeit less frequently and in more mild form [2,20]. The apparent increase in variance among lines can therefore be explained by Hsp90 buffering within-line rather than between-line variation. Our study avoids this problem by using statistical procedures that partition multiple contributions to variance.

Similarly to the MA lines, we found that GdA treatment tends to decrease phenotypic diversity among the Rec lines (Fig 5C). These differences between strain collections suggest that Hsp90’s influence on the amount of phenotypic variance between strains tends toward potentiation, but that natural selection tends to skew this genetic interaction toward one of buffering (Fig 5A–5D).

### Stabilizing Selection Can Transform Patterns of Line-Crossing Epistasis into Patterns with More Line Spreading

**Fig 6:**
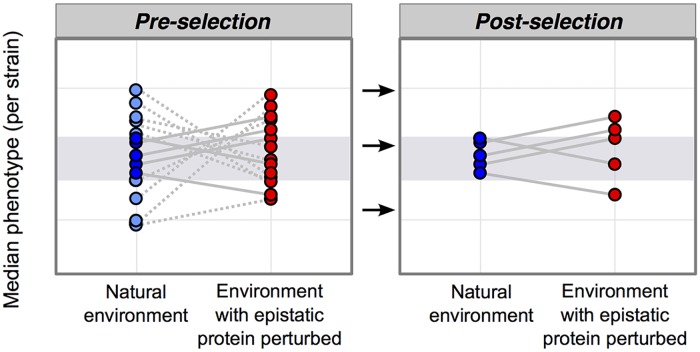
A model of how selection transforms the epistatic landscape, enriching for buffering. This model is schematized similarly to Fig 4A. Pre-selection: inhibiting an epistatic protein has genotype-specific effects on phenotype that result in line crossing. Stabilizing selection will narrow the amount of phenotypic diversity in the natural environment, removing genotypes with extreme phenotypes (light blue circles that fall outside of the grey shaded area). Removing these genotypes from the plot entirely (removing the light blue circles, dotted lines, and connected red circles) results in the post-selection plot. Post-selection: inhibiting an epistatic protein may reveal phenotypes outside of selection’s sieve (grey shaded area).

To further characterize how the relationship between Hsp90 and genetic variation differs in the MA and Rec lines versus the natural isolates, we partitioned genetic interactions into line spreading versus line crossing (see definitions in Fig 4A) for those phenotypes that had a significant interaction term in linear models (*p* < 0.01; orange shading in S1 Table). In all collections of yeast studied here, line spreading comprises only a relatively small percentage of Hsp90’s effect on phenotypic variation between strains, and, instead, line crossing dominates; this is especially true in the MA and Rec lines (Fig 5E). The selection hypothesis [7] explains how stabilizing selection can re-purpose epistatic proteins as buffers of standing genetic variation (Fig 6), transforming patterns dominated by line crossing (Fig 5E: MA and Rec lines) into patterns with more line spreading in the direction of buffering (Fig 5E: Ale, Div, SPH, and SPD strains).

## Discussion

For years, discussions of robustness and genetic canalization have featured Hsp90 [5,15,19–24]. The evidence that Hsp90 impairment reveals cryptic genetic variation fit a narrative in which mutationally robust genetic systems accumulate genetic variation that can be expressed when robustness is compromised [19,51]. Although the logic of this narrative is sound (decreasing robustness will reveal cryptic genetic variation), the converse (revelation of cryptic genetic variation means robustness has decreased) is not necessarily true. Indeed, as pointed out over a decade ago, revelation of cryptic genetic variation can occur even when robustness increases [7].

The persistence of the idea that Hsp90 is an agent of genetic canalization is especially unwarranted, because Hsp90 function has been shown in some cases to be required for new mutations to have effects [6,25,26]. As a result, two parallel descriptions of Hsp90 have existed: Hsp90 is a “capacitor” that buffers genetic variation until its function is impaired, and Hsp90 is a “potentiator” that enables new mutations to have effects [52,53]. When Hsp90 is viewed as both a capacitor and potentiator, the circularity of associating it with genetic canalization and mutational robustness should be even more apparent—Hsp90 buffers what it buffers (and does not buffer what it does not buffer).

Studies promoting the robustness hypothesis often reference Hsp90’s role as a protein-folding chaperone, painting a vivid picture of Hsp90 buffering mutational effects by helping proteins fold properly despite mutation [14,23]. But Hsp90 can influence mutant proteins indirectly, potentiating their effects by activating pathways that promote their expression [39] or activating stress response programs that allow mutant phenotypes to manifest without causing cell death [54]. Sometimes the contrasting roles of Hsp90 are not named as capacitance or potentiation, but rather are both attributed to Hsp90’s role in “buffering” mutant proteins, where the meaning of “buffering” is not limited to maintaining wild-type protein activity but also includes enabling new activity [26]. The confusion surrounding discussions of Hsp90's interactions with genetic variation could perhaps be cleared up by abandoning specialized terms such as "buffer" or "potentiator" in favor of descriptions of epistasis [10].

Our results provide direct evidence that Hsp90 does not tend to increase mutational robustness for the large set of cell-morphological traits we studied. Instead, the relationship between Hsp90 and new mutations is dominated by line-crossing epistasis. Therefore, on both theoretical and, now, empirical grounds, Hsp90 should not be described as an agent of genetic canalization. Using a very similar experimental design, except with deletion of the focal gene rather than pharmacological inhibition of its protein product, Richardson et al. (2013) reached the same conclusions about another candidate robustness-increasing factor, the histone variant H2A.Z. Together, the Hsp90 and H2A.Z results raise the question of whether any gene product increases robustness to mutation on a genome-wide scale [10].

Additionally, our results highlight that natural selection transforms the types of epistasis that are present in nature, leaving the false impression of robustness. Although it is appreciated that selection filters out certain types of new mutations (e.g., deleterious ones), the idea that natural selection also skews the types of epistasis that accumulate in genomes is much less appreciated. The false equality drawn between the genetic interactions common in nature and those that are common among new mutations has likely prevented a full and accurate understanding of natural variation in complex traits (including human disease traits).

To be clear, our results do not contradict the prevalence or importance of cryptic genetic variation in nature [55]. Our study confirms that natural genetic variation is enriched for buffered alleles. Previous work explains how the release of such variation, perhaps by stress that impairs Hsp90 function, may play a pivotal role in adaptation by revealing phenotypic diversity under novel or stressful conditions [1,2,5,56]. Revelation of cryptic genetic variation by environmental or genetic perturbation also likely contributes to complex human disease [13]. Our study contributes to previous work by clarifying why cryptic genetic variation exists, potentially shedding new light on the properties of cryptic alleles. For example, perhaps the biased subset of Hsp90-interacting variation that accumulates in nature tends to interact with Hsp90 via a different mechanism than most new mutations.

The nature of Hsp90’s interaction with new mutations is of high practical importance, because Hsp90 inhibitors are considered promising anticancer drugs. One rationale for inhibiting Hsp90 has been that Hsp90 protects tumor cells from the deleterious effects of their elevated mutation rates by suppressing the effects of many mutations [14]. Our work suggests that Hsp90 does not, on balance, protect cells from mutations’ effects and therefore challenges the validity of this rationale. An alternative and more straightforward rationale for inhibiting Hsp90 is that some Hsp90 client proteins are known to promote tumor development; targeting the chaperone therefore might work as a broad strategy for targeting signaling proteins to which cancer cells are “addicted” [15]. Although the rationale for this strategy does not depend on whether or not Hsp90 tends to suppress the effects of mutations, unfortunately, the strategy has not in general succeeded. The culprit appears to be that high doses of Hsp90 inhibitor are necessary, and these doses activate the stress-response factor HSF1, which itself promotes malignancy [15,26].

More recent work (upon which ongoing clinical trials are based) has focused on enhancing the efficacy of another anticancer drug by combining it with low-dose Hsp90 inhibition that does not activate the stress response [26]. The rationale in this case is that low-level inhibition is enough to prevent Hsp90 from potentiating the effects of adaptive mutations, thereby curtailing a tumor’s evolution of resistance to the other drug. A similar principle was proposed for the utility of Hsp90 inhibition in preventing resistance to antifungal drugs [25]. Results for estrogen receptor-positive breast cancer cells are promising [26]. It is possible that a bias toward potentiation of new mutations by Hsp90, as we observed in yeast, contributes to the success of such an approach. However, one might expect the contribution to success to be small, given the prevalence of line-crossing epistasis that we observed. Further investigation of the spectrum of effects of mutations that arise during adaptive evolution with and without Hsp90 inhibition, and ultimately the mechanism by which Hsp90 modifies the phenotypic effects of mutations, is warranted.

## Materials and Methods

### Yeast Growth, Staining, and Visualization

Yeast growth, cell staining, and microscopy were performed the same way for all six strain collections (for additional details about strain collections and sequencing of MA lines, see S1 Text as well as S3 Table). We prepared cells for microscopy using established protocols [9,38,57], with modifications (S1 Text). Briefly, yeast strains were grown from frozen stocks to saturation in rich media (YPD) in 96-well plates (48 h of growth). Each plate was used to inoculate a pair of new 96-well plates: a control plate containing synthetic complete media (SC) plus DMSO and an Hsp90-inhibited plate containing SC + 8.5 μM GdA solubilized in DMSO (Fig 2). Our goal was for everything about this pair of 96-well plates to be as similar as possible, including the concentration of DMSO, the identity of the yeast strain in each well, the orientation of plates during culturing, and the timing of all subsequent steps. This pair of 96-well plates (GdA+ and GdA−) was grown to saturation and then was used to inoculate a fresh pair of plates containing the same control or Hsp90-inhibited media (Fig 2A). This freshly inoculated pair of plates was grown for 6 h (to mid log), after which we removed growth media, added 4% paraformaldehyde, and fixed cells for 1 h. Cells were then stained overnight and mounted on 384-well glass bottom microscopy plates (Fig 2A). We performed epifluorescence microscopy using a Nikon Eclipse T*i* automated microscope using a 40× objective. For each strain, typically between 500 and 1,000 cells were imaged per condition (S1 Fig); at least two complete biological replicate experiments were performed per strain. Imaged cells were analyzed for quantitative morphological traits using CalMorph software (Fig 2B) [31].

### Pre-processing Cell Image Data

All data analysis was performed using the open-source R statistical computing package (http://www.r-project.org/), and analysis generally followed that done previously for similar morphology datasets [9,38,57], with modifications (S1 Text). Briefly, for each strain collection, each morphological trait was normalized to have a mean of zero and a standard deviation of one following Box-Cox transformation. Critically, this normalization allows the variances of GdA+ and GdA− conditions to differ, but standardizes the amount of variation across strain collections and phenotypes for downstream statistical analysis and visualization. Linear modeling was used to normalize data from replicate plates (S1 Text). As was done previously, we restricted our analysis to 132 high quality morphometric traits chosen for their lower replicate-to-replicate variation [38]. We eliminated redundancy among these traits by using PC analysis (PCA). The loadings of morphological traits onto PCs are fairly similar between strain collections (S1 Table).

### Detecting Genotype-By-GdA Interactions and Line Crossing versus Line Spreading

We used a linear model, fit using maximum likelihood in the R package lme4 [58], to estimate the contribution of genotype and condition to variation in each phenotype (PC). When a likelihood ratio test indicated that linear models including a genotype-by-condition interaction term fit the data significantly better than those without (*p* < 0.01), we reported a significant genotype-by-GdA interaction for that phenotype in that strain collection (orange shading in S1 Table). Partitioning of this interaction variance into line-crossing and line-spreading components (Figs 4B and 5E) was only done for PCs with a significant genotype-by-GdA interaction and was performed as described previously [9].

### Detecting Significant Differences in Between-Strain Variance upon Hsp90 Inhibition

For each PC, condition-specific strain means and between-strain variances were estimated from linear models using MCMC sampling with the R package MCMCglmm [37], following methodology outlined in a previous study (S1 Text) [9]. For each PC, the difference between the control and Hsp90-inhibited variances was called significant when the 95% highest posterior density interval of the difference obtained from the MCMC samples did not overlap zero. Although this analysis does not explicitly control for multiple hypothesis testing, the strong directionality of our results (i.e., that we detect seven and six PCs with decreased variance in MA and Rec lines, respectively, but none with increased variance) suggests that the trends we detect at this significance threshold are meaningful.

### Testing Effects of Less Growth versus Hsp90 Inhibition

We performed a control experiment on a subset of the MA lines, chosen randomly (21 MA lines; S2 Table), plus the MA line ancestor in order to directly compare the effects of 8.5 μM GdA, 5.0 μM radicicol, and a shortened exponential growth period. All experiments were performed using procedures described previously (Fig 2), except that all conditions (GdA, Rad, less growth) were present on the same 96-well plate. This plate was removed from the 30°C incubator once after 4 h to harvest cells in the “less growth” condition, and again after 6 h to harvest cells in other conditions; cells from all conditions were prepared for microscopy and imaged together (see S1 Text).

## Supporting Information

S1 Fig Extended information about the Hsp90-inhibited growth rates, cell-cycle phase, number, and strain identity of the cells imaged. **(A)** Growth rates were measured as log-linear increases in optical density over time. Each point represents growth of a single strain performed in a separate well on a 96-well plate; boxplots summarize the distributions of growth rates across all strains tested in a given condition displaying the median (center line), interquartile range (IQR) (upper and lower hinges), and highest value within 1.5 × IQR (whiskers). Representative strains from each collection were grown in SC with different concentrations of DMSO (blue) or geldanamycin (GdA) + DMSO (red). In all experiments, GdA is solubilized in DMSO, we therefore added DMSO to GdA− experiments such that the only difference between GdA+ and GdA− conditions is the presence/absence of GdA−. Fitness decreases along the horizontal axis in both the control and Hsp90-inhbited conditions because both GdA and DMSO reduce cell growth. We performed most experiments in 8.5 μM GdA as this concentration had only minimal effects on exponential growth rate relative to GdA−. The parents of the Rec cross are included among the Diverse strains surveyed (black outline: wine parent; tan outline: oak parent). **(B)** Bars represent the average proportion of cells in the unbudded stage of the cell cycle in our complete dataset. **(C)** Points represent the numbers of phenotyped cells that pass filtering for each strain in control and GdA-treated conditions. Boxplots represent the distributions of cell counts across all strains within a given collection and display the same summary statistics as **panel A**. **(D)** Bar heights represent the number of strains surveyed in a given collection.
(PDF)

S2 Fig GdA’s influence on the average phenotype of all 264 strains in this study, for each PC. These plots are similar to those in Fig 3A, except here all 6 PCs corresponding to unbudded cells (blue background), 9 PCS corresponding to cells with a small bud (green background) and 14 PCs corresponding to cells with a large bud (purple background) are shown. The vertical axis represents the average morphology of each yeast strain in the GdA− condition (left side of each plot) or the GdA+ condition (right side of each plot). The strain represented by a magenta rather than a black line corresponds to the ancestor of the MA lines, or in all other collections, the yeast strain with the response to GdA that is closest to the median response across all strains in that collection. This strain differs for each PC.
(PDF)

S3 Fig Several MA lines have unique responses to GdA, relative to the MA line ancestor. For each MA line and cell cycle phase, we measured the length of a single vector starting from the mean phenotypes in the GdA− condition and ending at the mean phenotypes in the GdA+ condition. The horizontal axis is the length (magnitude) of each vector, minus the length of the vector for the MA line ancestor. Then we converted all vectors to unit vectors by dividing all GdA− and GdA+ phenotypic means by the magnitude of the corresponding vector for each MA line. We shifted all unit vectors to begin at the origin and calculated the distance between the normalized GdA+ phenotypes for each MA line and the normalized GdA+ phenotypes for the ancestor (*i*.*e*. we calculated the distance between the end of the unit vector for each MA line and the end of the unit vector for the ancestor). This distance is plotted on the vertical axis. For the farthest points from the MA line ancestor, the name of the corresponding MA line is written on the plot.
(PDF)

S4 Fig The effects of radicicol, geldanamycin, and shortening the duration of exponential growth on average MA line morphologies. The structurally unrelated Hsp90 inhibitors GdA and Rad have similar effects on the morphologies of the MA lines, but the effect of modulating the length of exponential growth is not as similar. These plots are similar to those in Fig 4D except here plots for all 29 PCs are shown. The vertical axis represents the average morphology of each MA line in the 5.0 μM Rad condition (leftmost points in each plot), the 8.5 μM GdA condition (middle points in each plot) or the shortened growth condition (rightmost points in each plot). The Pearson Correlation Coefficient (***r***) is displayed for GdA vs. Rad (left side), and GdA vs. less growth (right side). PCs related to unbudded cells are highlighted in blue, small-budded cells in green, or large-budded cells in purple.
(PDF)

S5 Fig GdA’s divergent effect on morphological variation in strains experiencing reduced selection as compared to natural isolates is not driven by effects on growth. (A) The dots depict, for each of 132 morphological traits, changes to variance that occur across a subsample of 22 MA lines upon perturbing the length of exponential growth phase or treating cells with 8.5 μM GdA. Phenotypic variance between strains is quantified as it was for the full dataset, using MCMCglmm. Shortening the duration of exponential growth tends to increase between-strain variation in 132 morphological traits, while GdA treatment has a smaller and opposite effect. Boxplots summarize the distribution of these changes across all 132 traits after a given perturbation (light blue: less growth, black: 8.5 μM GdA). Boxplots display the median (center line), interquartile range (IQR) (upper and lower hinges), highest value within 1.5 × IQR (whiskers), and roughly a 95% confidence interval around the median calculated as 1.58 × IQR / √n (notches). (B) The effect of shortening exponential growth does not predict the effect of GdA treatment on between-strain variation. These points are the same as in panel A, but here, error bars represent the 95% posterior density interval surrounding the change in between strain variance for each of the 132 traits. Phenotypes are colored purple when, in the full dataset of MA lines, the difference in between-strain variance between GdA+ and GdA− conditions is significantly below zero. The phenotypes colored in purple are evenly spread along the horizontal axis suggesting the effect of GdA on morphological variation is not dependent on any concomitant effect of growth, at least not one that was captured by our particular growth manipulation. (C) Similar to Fig 5 except variance differences are shown for only 20 PCs drawn from 73/132 morphological phenotypes for which between strain variation is not significantly affected by the duration of exponential growth. (D) Similar to Fig 5 except here variance differences are plotted separately for those PCs corresponding to unbudded cells (6 PCs; 19 traits), small-budded cells (9PCs; 40 traits) and large-budded cells (14 PCs; 73 traits). (E) Similar to Fig 5 except here variance differences between only 20 MA lines that each possess a single coding mutation are plotted. In (C–E), boxplots are not notched as in Fig 5 and are always colored black.
(PDF)

S6 Fig Levels of morphological variation in collections of natural isolates suggest that morphological diversity has been constrained by selection. Each point represents a compromise PC and each boxplot summarizes the distribution across 51 compromise PCs for a single strain collection. Boxplots display the median (center line), interquartile range (IQR) (upper and lower hinges), highest value within 1.5 × IQR (whiskers), and roughly a 95% confidence interval around the median calculated as 1.58 × IQR / √n (notches). The vertical axis displays the amount of variance present between strains in the GdA− condition. For each PC, overall variance is scaled to 1 before variance is partitioned into within-strain, between-strain, GdA+, and GdA− components.
(PDF)

S1 Table Information about the 29 PCs included in this study including the percent of overall morphological variation explained by each PC, the top Calmorph morphological traits that load onto each PC, the effect of GdA on the variance of each PC, and whether position on a 96 well plate contributes significantly to variance of each PC. PCs are highlighted in orange corresponding to whether they are influenced by a GdA-by-genotype interaction at p < 0.01.
(XLSX)

S2 Table The identities of the single nucleotide mutations in each MA line plus information about which MA lines are the most responsive to GdA, relative to the ancestor. MA lines are highlighted in blue, green, or red corresponding to the color of that MA line in S3 Fig.
(XLSX)

S3 Table The collections of yeast strains surveyed in this study. (XLSX)

S1 Text (DOCX)

## Abbreviations

GdA: geldanamycin
MA: mutation accumulation
MCMC: Markov chain Monte Carlo
PC: principal component
PCA: principal component analysis
Rec: recombinant
