# Supplementary figures and images for: Selection Transforms the Landscape of Genetic Variation Interacting with Hsp90
Source: PLoS Biol. 2016 Oct 21;14(10):e2000465. doi: 10.1371/journal.pbio.2000465 (PMC5074785; doi:10.1371/journal.pbio.2000465)

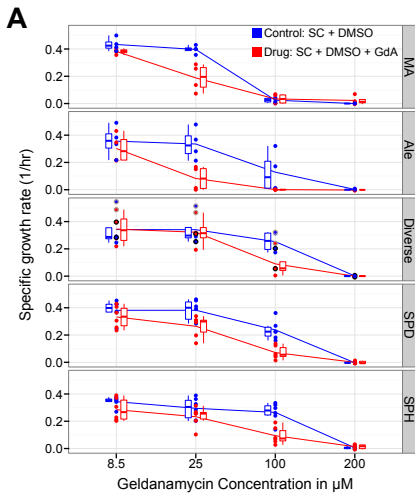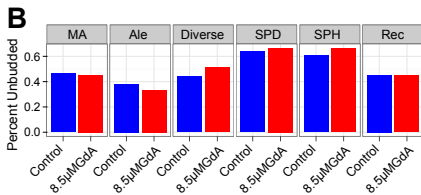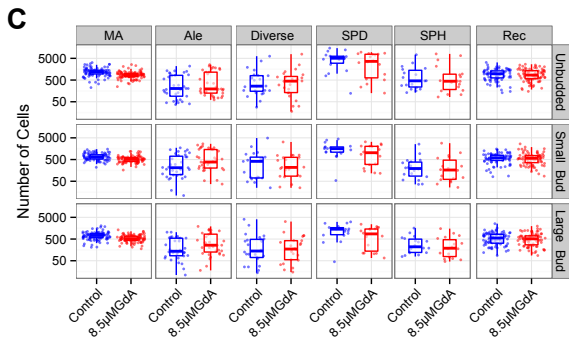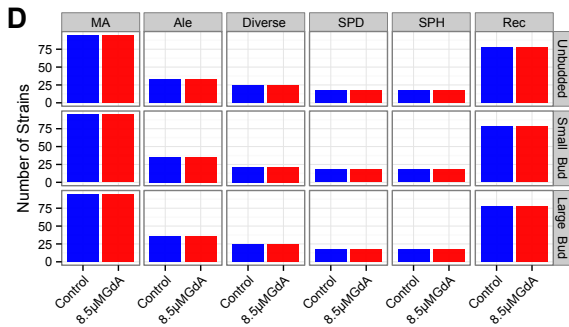

Supplement: S1 Fig — (A) Growth rates were measured as log-linear increases in optical density over time. Each point represents growth of a single strain performed in a separate well on a 96-well plate; boxplots summarize the distributions of growth rates across all strains tested in a given condition displaying the median (center line), interquartile range (IQR) (upper and lower hinges), and highest value within 1.5 × IQR (whiskers). Representative strains from each collection were grown in SC with different concentrations of DMSO (blue) or geldanamycin (GdA) + DMSO (red). In all experiments, GdA is solubilized in DMSO, we therefore added DMSO to GdA− experiments such that the only difference between GdA+ and GdA− conditions is the presence/absence of GdA−. Fitness decreases along the horizontal axis in both the control and Hsp90-inhbited conditions because both GdA and DMSO reduce cell growth. We performed most experiments in 8.5 μM GdA as this concentration had only minimal effects on exponential growth rate relative to GdA−. The parents of the Rec cross are included among the Diverse strains surveyed (black outline: wine parent; tan outline: oak parent). (B) Bars represent the average proportion of cells in the unbudded stage of the cell cycle in our complete dataset. (C) Points represent the numbers of phenotyped cells that pass filtering for each strain in control and GdA-treated conditions. Boxplots represent the distributions of cell counts across all strains within a given collection and display the same summary statistics as panel A. (D) Bar heights represent the number of strains surveyed in a given collection. (PDF) [file pbio.2000465.s001.pdf]

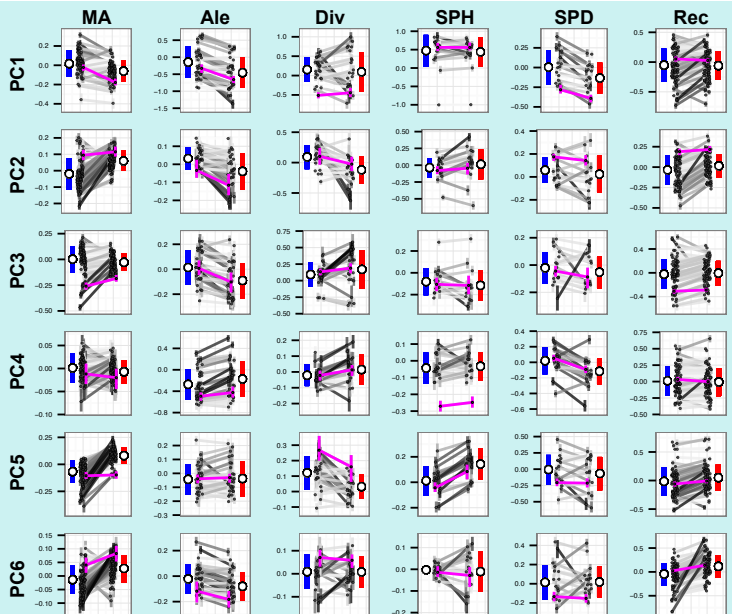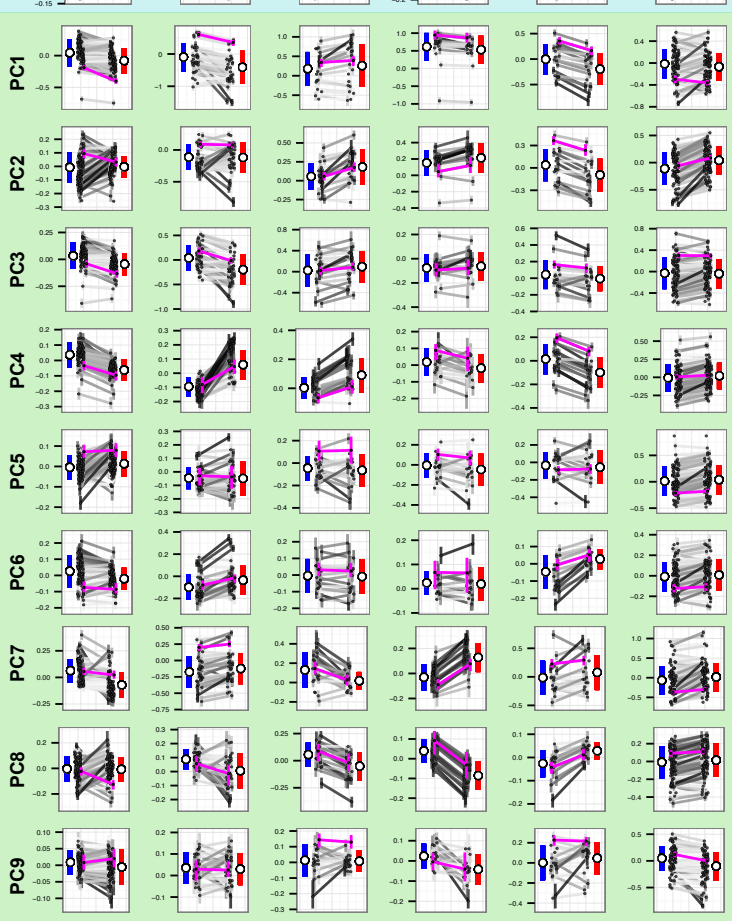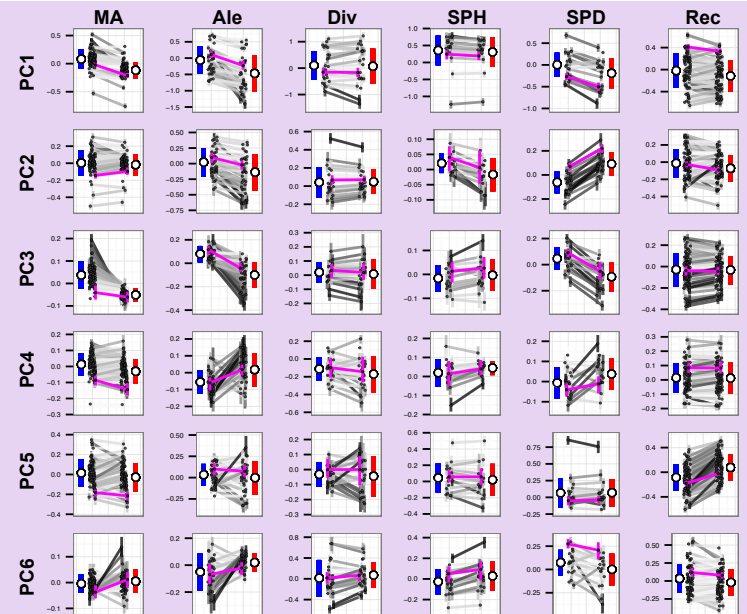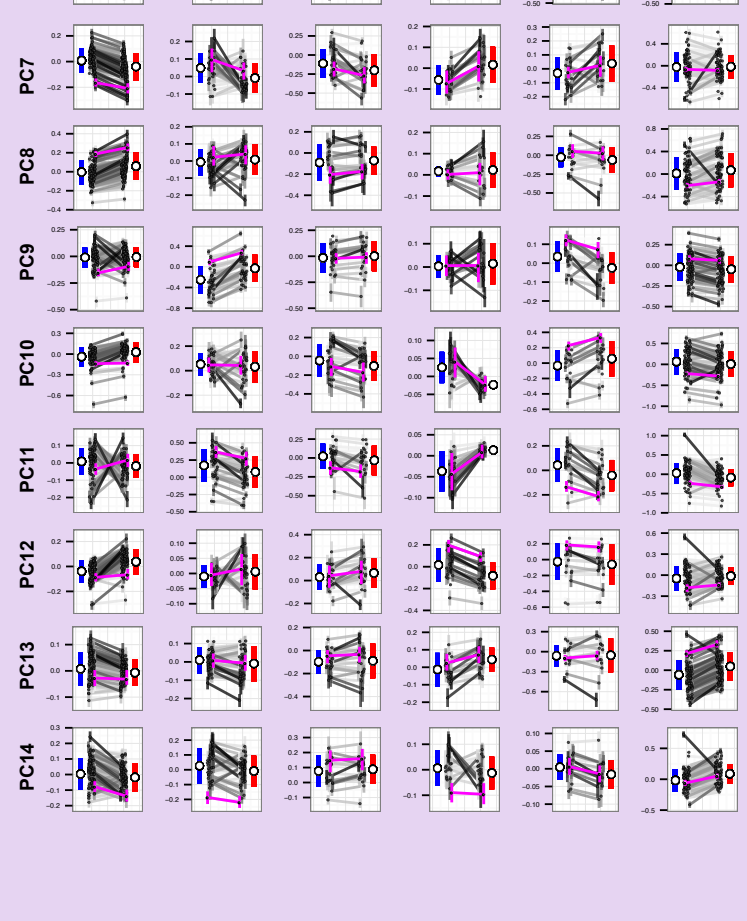

Supplement: S2 Fig — These plots are similar to those in Fig 3A, except here all 6 PCs corresponding to unbudded cells (blue background), 9 PCS corresponding to cells with a small bud (green background) and 14 PCs corresponding to cells with a large bud (purple background) are shown. The vertical axis represents the average morphology of each yeast strain in the GdA− condition (left side of each plot) or the GdA+ condition (right side of each plot). The strain represented by a magenta rather than a black line corresponds to the ancestor of the MA lines, or in all other collections, the yeast strain with the response to GdA that is closest to the median response across all strains in that collection. This strain differs for each PC. (PDF) [file pbio.2000465.s002.pdf]

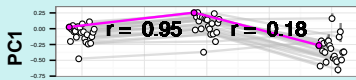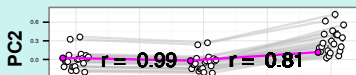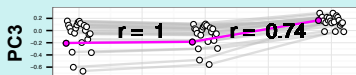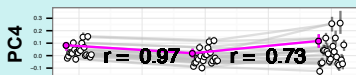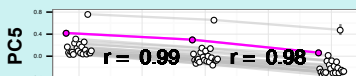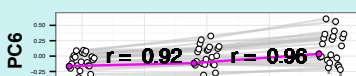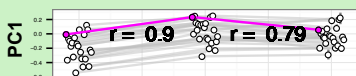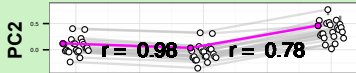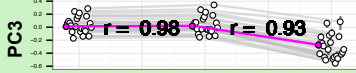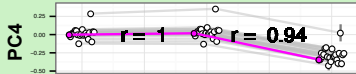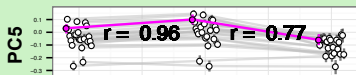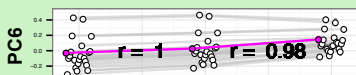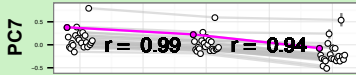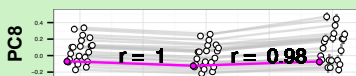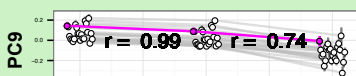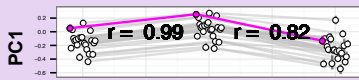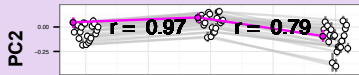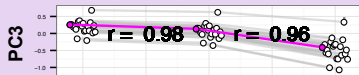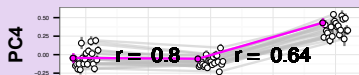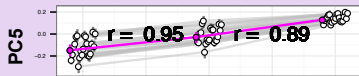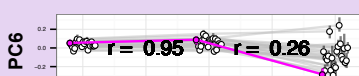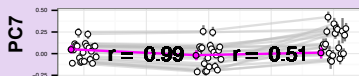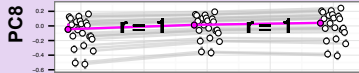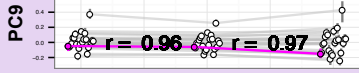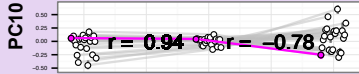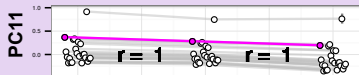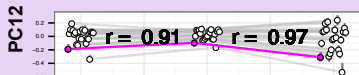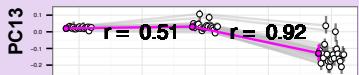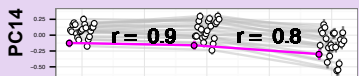

Supplement: S4 Fig — The structurally unrelated Hsp90 inhibitors GdA and Rad have similar effects on the morphologies of the MA lines, but the effect of modulating the length of exponential growth is not as similar. These plots are similar to those in Fig 4D except here plots for all 29 PCs are shown. The vertical axis represents the average morphology of each MA line in the 5.0 μM Rad condition (leftmost points in each plot), the 8.5 μM GdA condition (middle points in each plot) or the shortened growth condition (rightmost points in each plot). The Pearson Correlation Coefficient (r) is displayed for GdA vs. Rad (left side), and GdA vs. less growth (right side). PCs related to unbudded cells are highlighted in blue, small-budded cells in green, or large-budded cells in purple. (PDF) [file pbio.2000465.s004.pdf]

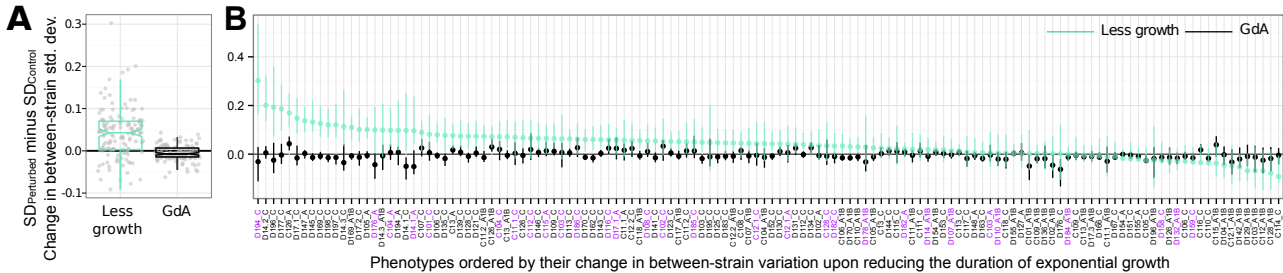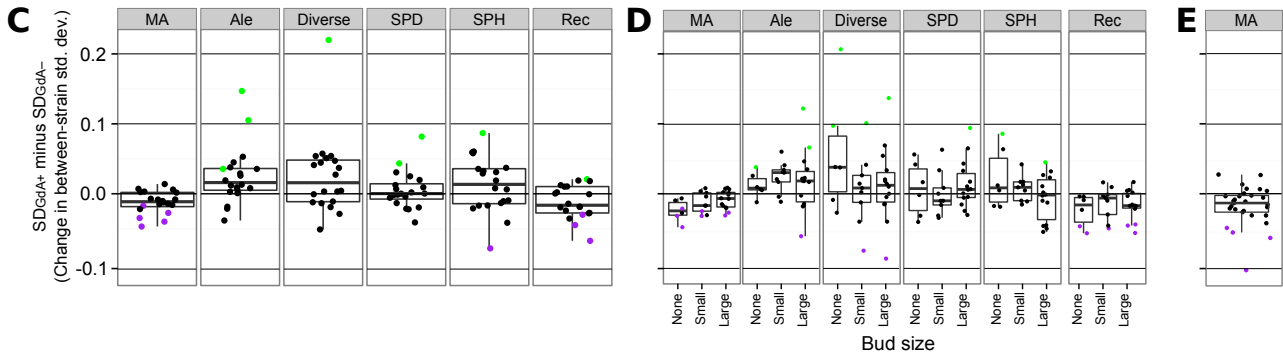

Supplement: S5 Fig — (A) The dots depict, for each of 132 morphological traits, changes to variance that occur across a subsample of 22 MA lines upon perturbing the length of exponential growth phase or treating cells with 8.5 μM GdA. Phenotypic variance between strains is quantified as it was for the full dataset, using MCMCglmm. Shortening the duration of exponential growth tends to increase between-strain variation in 132 morphological traits, while GdA treatment has a smaller and opposite effect. Boxplots summarize the distribution of these changes across all 132 traits after a given perturbation (light blue: less growth, black: 8.5 μM GdA). Boxplots display the median (center line), interquartile range (IQR) (upper and lower hinges), highest value within 1.5 × IQR (whiskers), and roughly a 95% confidence interval around the median calculated as 1.58 × IQR / √n (notches). (B) The effect of shortening exponential growth does not predict the effect of GdA treatment on between-strain variation. These points are the same as in panel A, but here, error bars represent the 95% posterior density interval surrounding the change in between strain variance for each of the 132 traits. Phenotypes are colored purple when, in the full dataset of MA lines, the difference in between-strain variance between GdA+ and GdA− conditions is significantly below zero. The phenotypes colored in purple are evenly spread along the horizontal axis suggesting the effect of GdA on morphological variation is not dependent on any concomitant effect of growth, at least not one that was captured by our particular growth manipulation. (C) Similar to Fig 5 except variance differences are shown for only 20 PCs drawn from 73/132 morphological phenotypes for which between strain variation is not significantly affected by the duration of exponential growth. (D) Similar to Fig 5 except here variance differences are plotted separately for those PCs corresponding to unbudded cells (6 PCs; 19 traits), small-budded cells (9PCs; [file pbio.2000465.s005.pdf]

Standard deviation between lines in GdA-condition

0.4  
0.3  
0.2  
0.1  
0.0

MA

Ale

Div

SPD

SPH

Rec

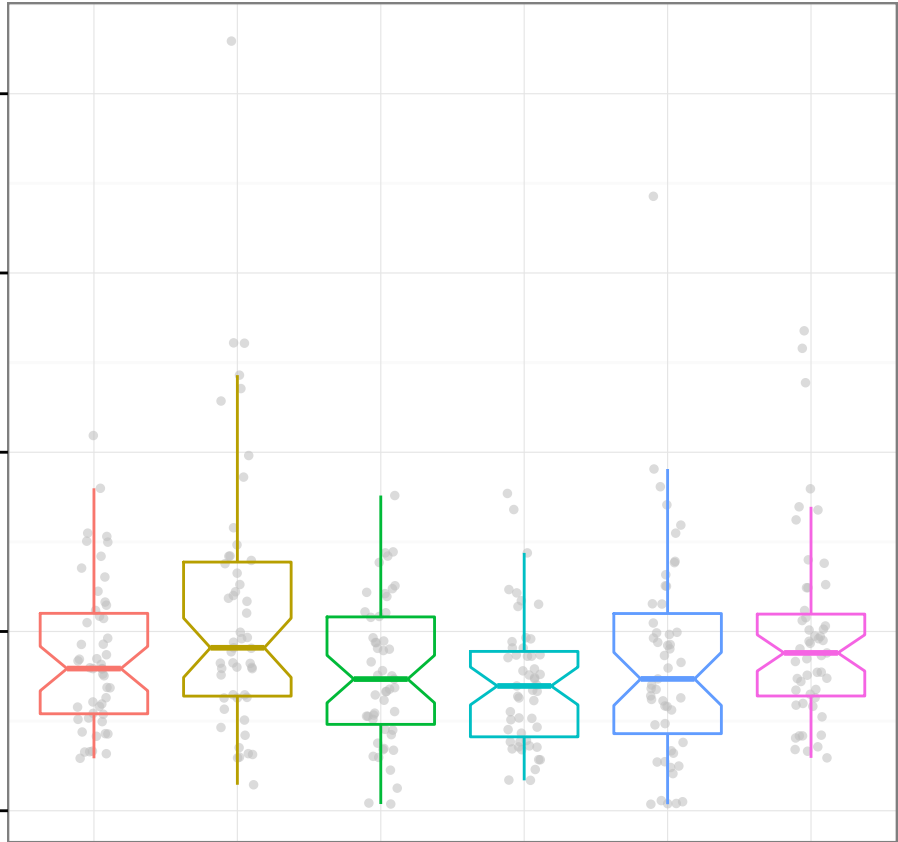

Supplement: S6 Fig — Each point represents a compromise PC and each boxplot summarizes the distribution across 51 compromise PCs for a single strain collection. Boxplots display the median (center line), interquartile range (IQR) (upper and lower hinges), highest value within 1.5 × IQR (whiskers), and roughly a 95% confidence interval around the median calculated as 1.58 × IQR / √n (notches). The vertical axis displays the amount of variance present between strains in the GdA− condition. For each PC, overall variance is scaled to 1 before variance is partitioned into within-strain, between-strain, GdA+, and GdA− components. (PDF) [file pbio.2000465.s006.pdf]
