# Supplementary material for: Selection Transforms the Landscape of Genetic Variation Interacting with Hsp90
Source: PLoS Biol. 2016 Oct 21;14(10):e2000465. doi: 10.1371/journal.pbio.2000465 (PMC5074785; doi:10.1371/journal.pbio.2000465)
Supplement: S3 Fig — For each MA line and cell cycle phase, we measured the length of a single vector starting from the mean phenotypes in the GdA− condition and ending at the mean phenotypes in the GdA+ condition. The horizontal axis is the length (magnitude) of each vector, minus the length of the vector for the MA line ancestor. Then we converted all vectors to unit vectors by dividing all GdA− and GdA+ phenotypic means by the magnitude of the corresponding vector for each MA line. We shifted all unit vectors to begin at the origin and calculated the distance between the normalized GdA+ phenotypes for each MA line and the normalized GdA+ phenotypes for the ancestor (i.e. we calculated the distance between the end of the unit vector for each MA line and the end of the unit vector for the ancestor). This distance is plotted on the vertical axis. For the farthest points from the MA line ancestor, the name of the corresponding MA line is written on the plot. (PDF) [file pbio.2000465.s003.pdf]

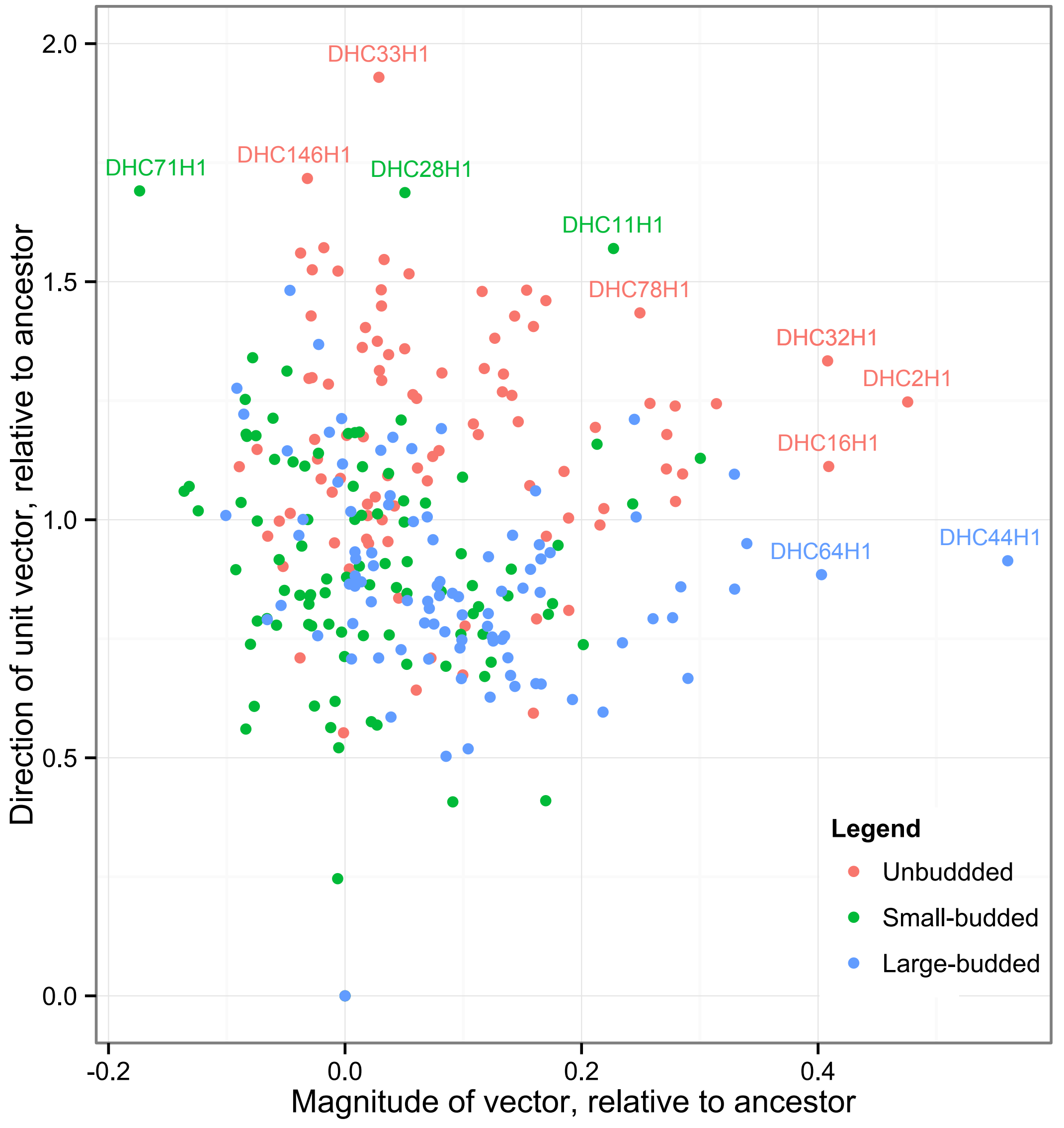

DHC33H1
DHC71H1
DHC146H1
DHC28H1
DHC11H1
DHC78H1
DHC32H1
DHC2H1
DHC16H1
DHC64H1
DHC44H1
2.0
1.5
1.0
0.5
0.0
-0.2
0.0
0.2
0.4
Direction of unit vector, relative to ancestor
Magnitude of vector, relative to ancestor
Legend
Unbuddded
Small-budded
Large-budded
