# Supplementary material for: Selection Transforms the Landscape of Genetic Variation Interacting with Hsp90
Source: PLoS Biol. 2016 Oct 21;14(10):e2000465. doi: 10.1371/journal.pbio.2000465 (PMC5074785; doi:10.1371/journal.pbio.2000465)
Supplement: S1 Text — (DOCX) [file pbio.2000465.s010.docx]

**Supplemental Experimental Procedures**

**Yeast strain collections**

We studied the following strain collections:

**1. MA lines:** The ‘MA line’ collection comprises 94 haploid derivatives of MA lines [1] that experienced decreased selection pressure [2]. In particular, selection on growth rate was reduced by propagating each MA line through single colonies that were randomly chosen, irrespective of colony size, based on proximity to an ‘X’ drawn onto plates [2]. The haploid derivatives of these MA lines that were used in this study are slightly different than those generated in a previous work [1], as previous haploid derivatives were suspected of having acquired [PSI+], the prion form of the translation-termination factor Sup35, which reverted an adenine auxotrophy that was present in the ancestor of all MA lines [1]. The lines in question have been replaced by new random haploid-derivative lines generated from the same diploids [1]. These lines have recently been sequenced [3] (**Table S2**). Most, but not all, MA lines in our study are of mating type **a***.* Restricting our analysis to the set of 84 haploids that are mating-type **a** produces very similar results to those reported in the main text.

**2. Rec lines:** The 78 ‘Rec lines’ are homothallic diploid progeny (“F2_1” through “F2_78”) from a mating between two natural isolates, one from wine fermentations and another from soil near an oak tree [4,5]. Pairwise divergence between these two isolates is about 0.006 SNPs per site [6].

**3. Ale strains:** The ‘ale strains’ include 36 commercially available polyploid strains used in ale production (Wyeast Laboratories, OR or White Labs, CA). The ale strains were chosen with the help of a local home brewing education and supply shop (Bitter & Esters, NY) in order to learn about their history and ensure that no duplicate strains were selected. We selected a single colony from each brewing yeast monoculture to study further. These strains likely experienced selection pressures associated with industrial fermentation. These strains have not been sequenced.

**4. Diverse strains:** The ‘diverse strains’ include 24 mating-type **a** haploid derivatives from a worldwide collection of yeast from diverse habitats [6,7]. We chose the 24 strains from this collection that were the least flocculent in order to capture images of single cells. These strains are very diverse; some are from wine fermentations, some are clinical isolates, and some are from soil environments. The average pairwise divergence among these strains is 0.0056 [6].

**5. SPD strains:** The ‘SPD strains’ include 18 diploid *S. paradoxus* strains collected from soil environments in two nearby parks in Great Britain [6,7]. The average pairwise divergence in this population is about 0.001 [6].

**6. SPH strains:** The ‘SPH strains’ include 18 haploid mating-type **a** derivatives of the diploid *S. paradoxus* strains described above.

**Yeast growth, staining, and visualization**

Growth, staining, and visualization were performed the same way for all six strain collections and all cultures were grown in a shaking incubator at 30ºC.

We prepared cells for microscopy using modified protocols that were originally developed in previous studies that also focused on measuring morphological variation in yeast [1,8,9]. Yeast strains were grown from frozen stocks to saturation in rich media (YPD) in 96-well plates. Each YPD plate was used to inoculate a pair of new 96-well plates: a control plate containing synthetic complete media (SC) + Dimethly sulfoxide (DMSO), and an Hsp90-inhibited plate containing SC + 8.5 μM GdA solubilized in DMSO (**Fig 2A**); this concentration of GdA has minimal effects on exponential growth rate (**Fig S1A**) and lag duration (**Fig S1B**). We added corresponding volumes of DMSO to control plates such that the only difference between GdA+ and GdA– plates is the presence or absence of GdA. Our goal was for everything about this pair of 96-well plates to be as similar as possible, including the identity of the yeast strain in each well, the orientation of plates during growths, and the timing of all subsequent steps.

This pair of 96-well plates (GdA+ and GdA–) was grown to saturation, and then was used to inoculate a fresh pair of plates containing the same control or Hsp90-inhibited media (**Fig 2A**). This freshly inoculated pair of plates was grown for 6 hours (to mid log) after which we removed growth media, added 4% paraformaldehyde, and fixed cells for 1 hour. Cells were then stained for cell-surface manno-protein using fluorescein isothiocyanate-Con A (Sigma) and nuclear DNA using 4′, 6-diamidino-2-phenylindole (Sigma) and cells were sonicated. Finally, paired 96-well plates were mounted side-by-side on 384-well glass bottom microscopy plates in Vectashield (Vector Laboratories) in duplicate (*i.e.* a 384 well plate has enough room to mount each 96 well plate twice) (**Fig 2A**).

We performed epifluorescence microscopy using a Nikon Eclipse T*i* automated microscope using a 40× objective. Over 100 micrographs were captured from each strain in each condition, typically yielding between 100 and 500 cell images per cell cycle stage, per replicate (**Fig S1C & S1D**). Imaged cells were analyzed for quantitative morphological traits using the CalMorph software package [10] (**Fig 2B**).

**Pre-processing cell image data**

All data analysis was performed using the open-source R statistical computing package (<http://www.r-project.org/>), and analysis generally followed that done previously for similar morphology datasets [1,8,9].

*Filtering cells*

For each morphological trait, cells representing extreme outliers (greater than 5 standard deviations from the mean) were removed from the dataset, as manual inspection typically revealed such cases to be debris or Calmorph miscalls. For the same reason, cells with severe deviations from an ellipsoid shape were also removed by screening phenotype C13 to exclude cells with values greater than 0.000017. Calmorph divides cells based on their stage of the cell cycle into three categories: unbudded, small-budded, and large-budded cells. For any cell stage of a given strain to be included in our study, we required a minimum of 10 cells each in the control and the Hsp90-inhibited conditions to pass this filtering. Typically far more cells than this minimum number passed filtering (**Fig S1C**), but for one yeast strain of the Diverse collection we obtained too few phenotyped cells of one particular phase (**Fig S1D**), so all cells of this strain were removed from the data matrix for the affected cell stage.

*Data transformation and accounting for replicate effects*

After filtering, data were structured into independent datasets for each strain collection (6 total: MA, Ale, Diverse, SPD, SPH, Rec). Each dataset contains GdA+ as well as GdA– conditions. Next, each Calmorph morphological trait in each dataset was normalized via a Box-Cox transformation to have a mean of zero and a standard deviation of one. Critically, this transformation allows the variances of GdA+ and GdA– conditions to differ, but normalizes the amount of variation across strain collections for downstream statistical analysis and visualization. Linear modeling was used to normalize data from replicate plates imaged on separate days by subtracting out plate effects. We fit linear mixed models using restricted maximum likelihood using the lme4 package in R. We model condition as a fixed effect and strain, replicate, as well as strain-by-condition, replicate-by-strain and replicate-by-condition interactions as random effects. We performed at least two complete biological replicates for all strain collections and imaged a minimum of 2 technical replicates of every strain in each condition per biological replicate experiment (*i.e.* there were a minimum of 2 replicate wells on each 384 well microscopy plate).

Previous work using this morphology assay did not suggest the presence of any obvious trends in yeast morphology resulting from positional effects. Nonetheless, we tested for positional effects by imaging a 96-well plate containing the same strain in each well. Linear modeling detected significant position effects across this 96-well plate for a minority of traits we study (43/132 single-cell morphological features and 9/29 principal components of morphology are affected by plate position at p<0.01; **Table S1**). For each yeast strain, cells grown in the control versus the GdA condition were mounted immediately next to each other (across columns; **Fig 2A**) in order to minimize any effect of distance that might exist.

*Filtering Calmorph traits and PCA*

We studied the same subset (132 traits) of the over 200 cell wall and nuclear traits that Calmorph can measure as was studied in previous research [9]. Morphological traits that were eliminated were those that are extremely noisy (typically phenotypes relating to the brightness of the cell or nuclear stain are very noisy) as well as those that were bimodal (and thus do not fulfill the requirements for statistical methods that assume normal distributions) and those that lacked values for the majority of cells (and thus were under-sampled relative to other traits).

Some of these 132 traits, such as cell area and long-axis length of unbudded cells, are correlated. We eliminated redundancy among phenotypes using principal component analysis (PCA). PCA was necessarily done separately for each of the three cell types (unbudded, small-budded, large-budded). We also chose to perform PCA separately for each strain collection because different strain collections might vary more in some phenotypes than in others. Nonetheless, the loadings of morphological traits onto PCs are fairly similar between strain collections (**Table S1**).

We initially selected as many PCs for each cell type as had a larger contribution to morphological variance than do PCs created from a randomly permutated dataset. However, there was slight variation in the number of significant PCs detected for each dataset (MA: 29, Ale: 26, Diverse: 26, SPD: 26, SPH: 24, Rec: 28). To simplify downstream analysis, we kept the same number of PCs for each strain collection (29 total) choosing the top 6 PCs representing 80 – 83% of the morphological variance in unbudded cells, 9 PCs representing 83 – 85% of the morphological variance in small budded cells, and 14 PCs representing 83 – 86% of the morphological variance in large budded cells (**Table S1**).

We rescaled each PC to have a mean of 0 and a variance of 1, which allowed us to more easily compare relative changes in variance across PCs.

**Estimation of condition-specific means and between-strain variances**

*Detecting significant genotype-by-GdA interactions*

We used a linear model, implemented using maximum likelihood in the R package lme4 [11], to estimate the contribution of genotype (modeled as a random effect) and condition (modeled as a fixed effect) to variation in each phenotype (PC). When a likelihood ratio test indicated that linear models including a genotype-by-condition interaction term fit the data significantly better than those without (p < 0.01), we reported a significant genotype-by-GdA interaction for that phenotype and strain collection.

*Implementing MCMCglmm*

Simpler linear models, such as the one described above, do not easily account for heterogeneous within-group variances [12], such as might be expected upon inhibiting Hsp90 [13]. Therefore, for each PC, condition-specific strain means and between-strain variances were estimated from linear models using Markov chain Monte Carlo sampling with the R package MCMCglmm [14], following methodology outlined in a previous study [1]. Our linear model specified two within-strain variances, one each for the GdA+ and the GdA– conditions, using the ‘‘idh’’ variance structure for the residual variance. Similarly, it specified two between-strain variances, and also a genetic covariance, using the ‘‘us’’ variance structure. As in previous work [1], Markov chains were run with a burn-in period of 6000, and samples were stored at intervals of 15 iterations for 30000 total iterations. This procedure was performed separately for all 29 PCs in each strain collection. Chain lengths were kept relatively short because of the large number of models that were run; previous work demonstrated that, in a similar dataset, longer chain lengths did not make a difference [1].

These linear models estimate strain means as well as between-strain variances.

*Detecting significant differences in between-strain variance upon Hsp90 inhibition*

For each PC, the difference between the control and Hsp90-inhibited variances was called significant when the 95% highest posterior density interval of the difference obtained from the MCMC samples did not overlap zero. The overall trend among all 29 PCs was called significant when the 95% confidence interval surrounding the median value did not overlap zero. The 95% confidence interval around the median was calculated as 1.58 × interquartile range / √n.

We choose a 95% significance threshold because detecting significant differences in variance requires more power than detecting significant differences in mean [15]. Despite multiple testing concerns, the strong directionality of our results (*e.g.* that we detect 7 and 6 PCs with decreased variance in MA and Rec lines respectively, but none with increased variance) suggests that the trends we detect at this significance threshold are meaningful.

*Quantifying line spreading versus line crossing*

Partitioning of interaction variance into line-crossing and line-spreading components (**Fig4B**, & **Fig 5E**) was only done for PCs with a significant genotype-by-GdA interaction term in linear models (p < 0.01) and was performed as described previously [1].

**Measuring Hsp90-sensitivity in MA lines**

*Quantifying MA Line responses to GdA*

We used the R package MCMCglmm [14] to estimate the average value and standard deviation of each yeast strain we study, for each PC, in the GdA– and GdA+ condition (grey/black open circles and vertical bars in **Fig 3A**, **Fig 4C**, **Fig 5D** & **Fig S2**). For each PC, we quantify an MA line’s response to GdA treatment as the change in a given MA line’s phenotype upon GdA treatment, relative to that of the MA ancestral strain.

*Detecting MA lines possessing Hsp90-sensitive mutations*

Our observations suggest that multiple MA lines possess mutations that interact (either directly or indirectly) with Hsp90 (**Fig 3**). Determining exactly which MA lines possess these mutations is outside the scope of our study, however, the following analyses may be of use to future researchers interested in exploring this question.

For each MA line, we calculated the 99% posterior density interval surrounding the difference in MA line versus ancestral response to GdA. We report, per MA line, the number of PCs for which this interval does not include zero in **Table S2** (column 12). We also report the number of PCs for which an MA line’s response is the most divergent from the ancestor, i.e. more divergent than the other 93 lines (**Table S2**; column 13). Because there are 29 PCs, this can happen for a maximum of 29 lines, and does so for 17 lines (**Fig 3B**) at a maximum rate of 6 times per line (**Table S2**; column 13).

Additionally, for each MA line we summarized the magnitude of the change in morphology and the direction of this change in the space of PCs related to a given phase of the cell cycle (**Fig S3; Table S2**). For each MA line and cell cycle phase, we measured the length of a single vector starting from the mean phenotypes in the GdA– condition and ending at the mean phenotypes in the GdA+ condition. The horizontal axis in **Fig S3** is the length (magnitude) of each vector, minus the length of the vector for the MA line ancestor. Then we converted all vectors to unit vectors by dividing all GdA­– and GdA+ phenotypic means by the magnitude of the corresponding vector for each MA line. We shifted all unit vectors to begin at the origin and calculated the distance between the normalized GdA+ phenotypes for each MA line and the normalized GdA+ phenotypes for the ancestor (*i.e.* we calculated the distance between the end of the unit vector for each MA line versus the end of the unit vector for the ancestor). This distance represents how much the direction of a given MA line’s response to GdA differs from the ancestor and is plotted on the vertical axis in **Fig S3**. MA lines that differ most strongly from the ancestor in either magnitude or direction are labeled on **Fig S3**.

*Sequencing and identifying single nucleotide mutations in the haploid MA lines*

94 haploid MA lines were cultured overnight in 3 ml YPD. Whole-genome DNA samples were extracted and sequenced as 100-bp paired-end Illumina multiplexed libraries on a single lane. Reads were mapped to the previously identified MA ancestral genome with bwa v0.5.9 [16], samtools v0.1.18 [17], and picard tools v1.55. Duplicated read pairs were masked and resulting reads locally realigned with GATK v2.1-8 [18]. Because the parental diploid single nucleotide mutations (SNMs) for all strains were known, we focused on the presence and absence of these known variants in the resulting haploids. SNMs were called as present if >90% of at least 4 reads supported the presence of the known variant, absent if >90% of at least 4 reads supported the presence of the reference allele, and NA if too few reads covered the position. Most such assignments were trivial due to haploid nature and prior knowledge of variants. The mutations were then assigned SIFT scores to gauge their effects on protein folding [19]. Due to random assortment, SNMs were only confirmed as inherited in 69 of the 94 haploid MA lines (**Table S2**); other lines might have SNMs in difficult to sequence regions, and the possibility of a non-trivial number of mutations in simple sequence repeats cannot be ruled out [20].

**Testing the effect of GdA on growth-related phenotypes**

*Exponential growth rate*

Choosing an appropriate concentration of Hsp90 inhibitory drug is challenging. On one hand, detecting significant differences in variance is difficult [15], so it is tempting to choose higher concentrations of GdA in order to maximize its effect on morphological phenotypes. On the other hand, inhibiting Hsp90 by any means, either using a drug or a genetic modification, can result in decreased growth rate and induction of stress response proteins [21], both of which may have independent effects on yeast morphology. Previous budding yeast studies used GdA concentrations ranging from 5 µM [21] to 200 µM [13]. We tested the effects of several GdA concentrations on yeast’s exponential growth rate.

In our experiments testing the effect of GdA on growth rate (**Fig S1A**), growth conditions were exactly the same as for our microscopy experiments: saturation in 96 well plates in YPD, followed by saturation in SC + DMSO with or without GdA, followed by exponential growth in SC + DMSO with or without GdA (**Fig 2A**). During exponential growth, optical density measurements were recorded every 15 minutes using a Tecan Infinite plate reader. Unlike in microscopy experiments, exponential growth was allowed to proceed to saturation. Growth rates were measured as maximum log-linear increases in optical density over time. We performed such measurements on a subset of strains in each collection, including 2 MA lines (each with a replicate), 3 ale strains (one with a replicate), 4 diverse strains, 6 SPD and 6 SPH strains (**Table S3**). Although we did not perform these measurements on the Rec lines, we did so on the diverse lines that were mated in order to generate the Rec lines (highlighted points in **Fig S1A**).

Of the GdA concentrations we tested, most have a strong effect on yeast exponential growth rate. High concentrations of DMSO also appear to affect yeast exponential growth rate; growth rate in both the GdA+ and GdA– conditions declines across the horizontal axis of **Fig S1A**. However, 8.5 µM GdA appears to have a minimal effect on growth as compared to the control condition. We therefore performed our experiments in this concentration of GdA.

*Lag duration*

We wondered whether any effect of GdA on lag duration might influence our quantification of morphological variation between yeast strains. Consider the simple case in which 8.5 µM GdA significantly increases lag phase to six hours. Six hours after transferring saturated cultures to fresh media (**Fig 2A**), cells in the GdA+ condition would still be in the unbudded phase of the cell cycle, while cells in GdA­– condition might appear more diverse because many would be actively budding and dividing. Perhaps, this could influence our estimation of the morphological variation between yeast strains in the GdA– versus the GdA+ conditions.

One way to estimate the length of lag duration is to quantify the proportion of cells in the unbudded phase of the cell cycle. This proportion does not appear to be influenced by GdA in a consistent way across all six strain collections, but perhaps is influenced in a collection-specific way (**Fig S1B**). To test this, we performed linear modeling, first transforming the proportion of unbudded cells per strain using an arcsin square-root transformation. We modeled the effect of GdA as a fixed effect, the effect of strain collection as a random effect, and the GdA-by-collection interaction term as a random effect. Linear modeling does not detect significant effects of GdA on the proportion of unbudded cells, or a significant collection-by-GdA interaction (p > 0.1).

Other factors than lag might influence the proportion of unbudded cells, so we re-examined our results to look at single-cell phenotypes relating to cells that are actively budding. Our morphometric analysis assigns cells to groups based on their bud size (*i.e.* unbudded, small-budded, and large-budded) [10]. If we focus on phenotypes that are only measured in large- or small-budded cells (cells that are out of lag), we see similar effects of GdA on between-strain variation as in the full dataset (**Fig S5D**). This suggests that the effect of GdA on between-strain variation does not result from its effects on lag duration.

**Comparing the effects of unrelated Hsp90 inhibitors (GdA and Rad) to the effects of altering the duration of exponential growth**

We performed a control experiment on a subset of the MA lines, chosen randomly (21 MA lines; Table S2), plus the MA line ancestor in order to directly compare the effects of 8.5 µM GdA, 5.0 µM radicicol, and a shortened growth period. Radicicol was dissolved such that the same volume of DMSO was added to GdA+ media, Rad+ media and control media. All experiments were performed using procedures described previously (**Fig 2**), except for this experiment multiple conditions (control, GdA, Rad, Less growth) were present on the same 96 well plate. This plate was removed from the 30ºC incubator once after 4 hours to harvest cells in the ‘less growth’ condition, and again after 6 hours to harvest cells in other conditions.

We used the R package MCMCglmm [14] to estimate the average value and standard deviation of each MA line in each of the conditions, for each PC, (grey/black open circles and vertical bars in **Fig 4D** & **Fig S4**). We measured whether these conditions have similar effects by calculating the Pearson correlation coefficient (***r*** in **Fig 4D** & **Fig S4**) between MA line morphologies in each condition. The excellent correlation we observe between the effects of 8.5 µM GdA and 5.0 µM Rad for most phenotypes (**Fig S4**), given that 5.0 µM Rad does not induce proteins associated with the general stress response [21], suggests that neither drug’s effects on morphology are mediated through induction of a stress response. We also used MCMCglmm to estimate the between-strain variances in multiple conditions; we found that perturbing the length of exponential growth sometimes influences the amount of morphological variation between strains, but not in a way that explains the effects of GdA on between-strain variance (**Fig S5A**, **Fig S5B** & **Fig S5C**).

**Comparing the amount of morphological variation between the six strain collections**

In order to compare the amount of morphological diversity across strain collections in the GdA– condition (**Fig S6**), we performed statistical analysis that differs from that described above in several important ways. Data from each strain collection were not treated separately because standardizing each dataset to have a mean of 0 and standard deviation of 1 would obscure differences between datasets. However, by studying all strain collections at once, we introduced concerns that properties of one collection would dominate over others creating biases that might affect Box-Cox transformation and selection of PCs.

To choose an unbiased value of lambda (*i.e.* the power to which each raw data value should be raised) for Box-Cox transformation, we used the EnvStats package [22] in R to estimate the transformation of the raw data from the residuals of a linear model where strain is modeled as a fixed effect. To select unbiased PCs, we utilized Dual Multiple Factor Analysis (Dual MFA), a variant of PC analysis adapted for structured datasets [23]. We chose 51 compromise PCs that each represent greater than 1% of the variance in unbudded, small-budded, or large-budded cells.

Across 51 compromise PCs, the level of morphological diversity among MA and Rec lines is very similar to that in the four other strain collections representing natural yeast isolates (compare boxplots in **Fig S6**). Because the amount of genetic variation in collections of natural isolates far exceeds that in the MA lines [6], their similar levels of morphological diversity suggests that phenotypic variation in single cell morphology is constrained by selection. This observation is consistent with the findings from previous studies of morphological diversity in the Diverse collection [24]. Although the Ale lines possess the most between-line morphological diversity relative to other collections, it is unlikely that these lines have escaped selection given the strict procedures under which brewers choose and perpetuate yeast with specific growth rates and flocculation properties. We suspect the Ale strains are more phenotypically variable than other natural isolates because they possess variation in ploidy, whereas other collections studied here do not.

**Supplemental References**

1. Richardson JB, Uppendahl LD, Traficante MK, Levy SF, Siegal ML. Histone variant HTZ1 shows extensive epistasis with, but does not increase robustness to, new mutations. Petrov DA, editor. PLoS Genet. 2013;9: e1003733. doi:10.1371/journal.pgen.1003733

2. Hall DW, Mahmoudizad R, Hurd AW, Joseph SB. Spontaneous mutations in diploid Saccharomyces cerevisiae: another thousand cell generations. Genet Res (Camb). Cambridge University Press; 2008;90: 229–241. doi:10.1017/S0016672308009324

3. Zhu YO, Siegal ML, Hall DW, Petrov DA. Precise estimates of mutation rate and spectrum in yeast. Proc Natl Acad Sci USA. National Acad Sciences; 2014;111: E2310–8. doi:10.1073/pnas.1323011111

4. Gerke J, Lorenz K, Cohen B. Genetic interactions between transcription factors cause natural variation in yeast. Science. American Association for the Advancement of Science; 2009;323: 498–501. doi:10.1126/science.1166426

5. Gerke JP, Chen CTL, Cohen BA. Natural isolates of Saccharomyces cerevisiae display complex genetic variation in sporulation efficiency. Genetics. Genetics Society of America; 2006;174: 985–997. doi:10.1534/genetics.106.058453

6. Liti G, Carter DM, Moses AM, Warringer J, Parts L, James SA, et al. Population genomics of domestic and wild yeasts. Nature. 2009;458: 337–341. doi:10.1038/nature07743

7. Cubillos FA, Louis EJ, Liti G. Generation of a large set of genetically tractable haploid and diploid Saccharomyces strains. FEMS Yeast Res. Blackwell Publishing Ltd; 2009;9: 1217–1225. doi:10.1111/j.1567-1364.2009.00583.x

8. Levy SF, Siegal ML. Network hubs buffer environmental variation in Saccharomyces cerevisiae. Levchenko A, editor. PLoS Biol. Public Library of Science; 2008;6: e264. doi:10.1371/journal.pbio.0060264

9. Bauer CR, Li S, Siegal ML. Essential gene disruptions reveal complex relationships between phenotypic robustness, pleiotropy, and fitness. Mol Syst Biol. 2015;11: 773–773. doi:10.15252/msb.20145264

10. Negishi T, Nogami S, Ohya Y. Multidimensional quantification of subcellular morphology of Saccharomyces cerevisiae using CalMorph, the high-throughput image-processing program. J Biotechnol. 2009;141: 109–117. doi:10.1016/j.jbiotec.2009.03.014

11. Bates D, Mächler M, Ben Bolker, Walker S. Fitting Linear Mixed-Effects Models Using lme4. Journal of Statistical Software. 2015;67: 1–48. doi:10.18637/jss.v067.i01

12. Geiler-Samerotte KA, Bauer CR, Li S, Ziv N, Gresham D, Siegal ML. The details in the distributions: why and how to study phenotypic variability. Curr Opin Biotechnol. 2013;24: 752–759. doi:10.1016/j.copbio.2013.03.010

13. Hsieh Y-Y, Hung P-H, Leu J-Y. Hsp90 regulates nongenetic variation in response to environmental stress. Mol Cell. 2013;50: 82–92. doi:10.1016/j.molcel.2013.01.026

14. Hadfield JD. MCMC methods for multi-response generalized linear mixed models: the MCMCglmm R package. Journal of Statistical Software. 2010.

15. Rönnegård L, Valdar W. Recent developments in statistical methods for detecting genetic loci affecting phenotypic variability. BMC Genet. BioMed Central Ltd; 2012;13: 63. doi:10.1186/1471-2156-13-63

16. Li H, Durbin R. Fast and accurate long-read alignment with Burrows-Wheeler transform. Bioinformatics. 2010;26: 589–595. doi:10.1093/bioinformatics/btp698

17. Li R, Li Y, Fang X, Yang H, Wang J, Kristiansen K, et al. SNP detection for massively parallel whole-genome resequencing. Genome Res. 2009;19: 1124–1132. doi:10.1101/gr.088013.108

18. McKenna A, Hanna M, Banks E, Sivachenko A, Cibulskis K, Kernytsky A, et al. The Genome Analysis Toolkit: a MapReduce framework for analyzing next-generation DNA sequencing data. Genome Res. Cold Spring Harbor Lab; 2010;20: 1297–1303. doi:10.1101/gr.107524.110

19. Kumar P, Henikoff S, Ng PC. Predicting the effects of coding non-synonymous variants on protein function using the SIFT algorithm. Nat Protoc. 2009;4: 1073–1081. doi:10.1038/nprot.2009.86

20. Lynch M, Sung W, Morris K, Coffey N, Landry CR, Dopman EB, et al. A genome-wide view of the spectrum of spontaneous mutations in yeast. Proc Natl Acad Sci USA. National Acad Sciences; 2008;105: 9272–9277. doi:10.1073/pnas.0803466105

21. Jarosz DF, Lindquist S. Hsp90 and environmental stress transform the adaptive value of natural genetic variation. Science. American Association for the Advancement of Science; 2010;330: 1820–1824. doi:10.1126/science.1195487

22. Millard SP. EnvStats. New York, NY: Springer Science & Business Media; 2013. doi:10.1007/978-1-4614-8456-1

23. Abdi H, Williams LJ, Valentin D. Multiple factor analysis: principal component analysis for multitable and multiblock data sets. WIREs Comp Stat. John Wiley & Sons, Inc; 2013;5: 149–179. doi:10.1002/wics.1246

24. Yang M, Ohnuki S, Ohya Y. Unveiling nonessential gene deletions that confer significant morphological phenotypes beyond natural yeast strains. BMC Genomics. 2014;15: 932. doi:10.1186/1471-2164-15-932
